# Correction: Extracellular Vesicle-Mediated Transfer of Genetic Information between the Hematopoietic System and the Brain in Response to Inflammation

**DOI:** 10.1371/journal.pbio.1002623

**Published:** 2018-03-12

**Authors:** The PLOS Biology Staff

## Notice of republication

This article was republished on August 25, 2015, to correct an error in the display of the sixth author's name. Author Domenico Del Turco was erroneously noted as Turco DD, rather than Del Turco, D. The publisher apologizes for the error. Please download this article again to view the correct version. The originally published, uncorrected article is provided here for reference.

## Supporting information

S1 File Originally published, uncorrected article. (PDF)
