# Supplementary figures and images for: Extracellular Vesicle-Mediated Transfer of Genetic Information between the Hematopoietic System and the Brain in Response to Inflammation
Source: PLoS Biol. 2014 Jun 3;12(6):e1001874. doi: 10.1371/journal.pbio.1001874 (PMC4043485; doi:10.1371/journal.pbio.1001874)

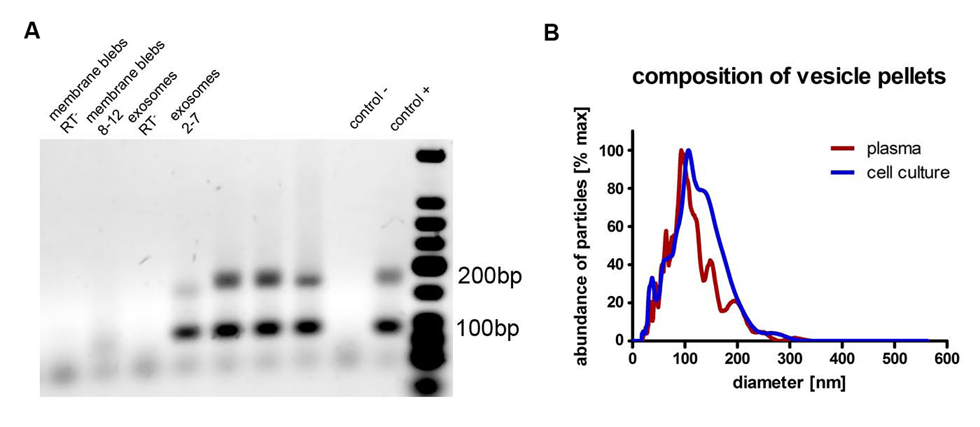

Supplement: Figure S1 — Analysis of exosomes derived from Vav1-Cre mice. (A) In this analysis, Cre mRNA is only present in the pooled exosomal fractions 2–7. The nonexosomal vesicle fractions (8–12) contain no detectable amounts of Cre mRNA. (B) Nanosight analysis of vesicle size distribution in exosomes isolated from blood plasma (in red) or from the supernatant of cultivated blood cells (in blue) from Vav1-iCre mice. An increase in larger size vesicles can be observed for the cell culture supernatant-derived EVs, indicating an increase in membrane blebs/apoptotic bodies or clustering of vesicles in culture. (TIF) [file pbio.1001874.s001.tif]

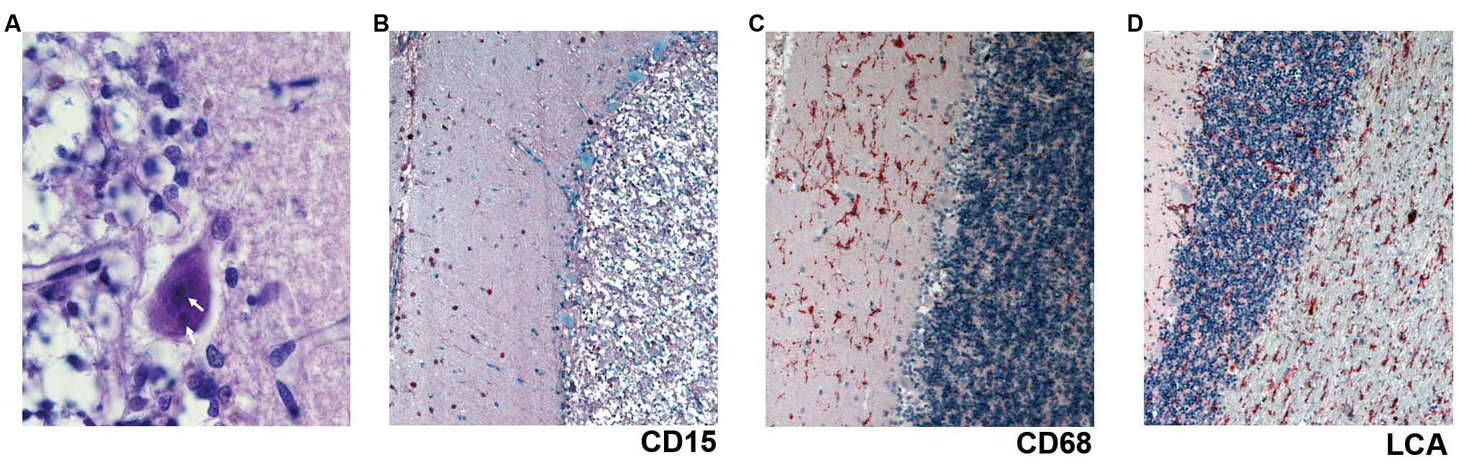

Supplement: Figure S2 — No evidence for elevated numbers of binucleated Purkinje neurons in cerebella of human patients with inflammation. (A) Rare occurrence of a second nucleus-like inclusion in a Purkinje neuron of a human cerebellum from a patient with a diagnosed inflammation (n<4 for all neurons in all analyzed cases). In these rare cases, the possible nuclear structures were all of very small size (4 µm) and clearly distinct from the large neuronal nuclei typical for Purkinje neurons. Purkinje neurons containing two similar neuronal nuclei indicating heterotypic cell fusion and subsequent reprogramming of the hematopoietic nucleus were never observed. Archived brain tissue of each patient was tested for inflammatory lesions with the combination of markers CD15 for granulocytes (B), CD68 for activated microglia (C), and LCA for mononuclear leucocytes (D). (TIF) [file pbio.1001874.s002.tif]

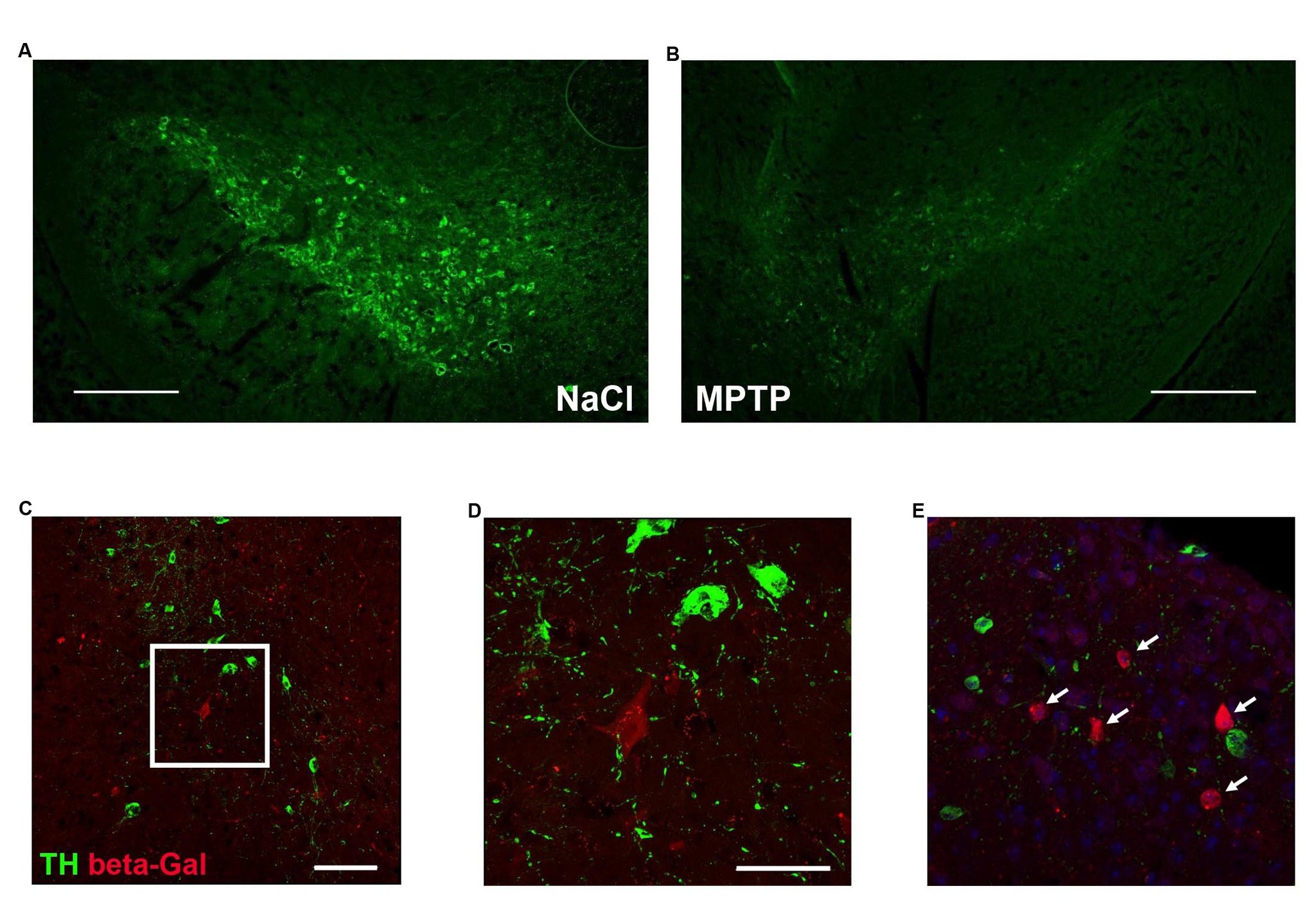

Supplement: Figure S3 — Frequent occurrence of recombined TH-negative cells with neuronal morphology in the SN/VTA in the MPTP mouse model of Parkinson's disease. Reduction in the number of TH-positive cells in the SN after MPTP treatment (B) as compared to NaCl (A). (C and D) TH-negative cell with neuronal morphology next to TH-positive dopaminergic neurons in the SN. (E) Several TH-negative cells with neuronal morphology expressing the reporter gene in the midbrain in brains of MPTP-treated mice. (TIF) [file pbio.1001874.s003.tif]

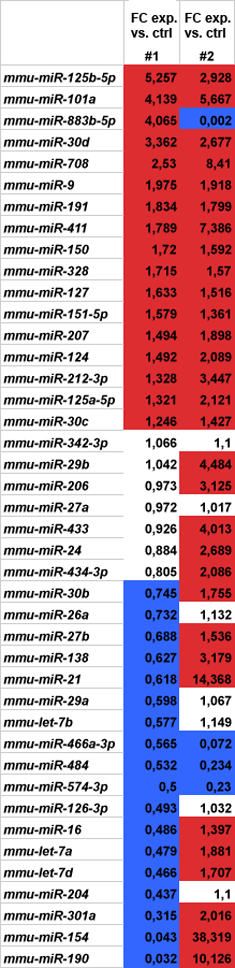

Supplement: Figure S4 — Heatmap of miRNA expression in recombined–nonrecombined pairs of Purkinje neurons. Linear fold changes between recombined and nonrecombined neurons are listed. Values >1.2 and <0.83 are colored red and blue, respectively. (TIF) [file pbio.1001874.s004.tif]
