# Supplementary material for: Extracellular Vesicle-Mediated Transfer of Genetic Information between the Hematopoietic System and the Brain in Response to Inflammation
Source: PLoS Biol. 2014 Jun 3;12(6):e1001874. doi: 10.1371/journal.pbio.1001874 (PMC4043485; doi:10.1371/journal.pbio.1001874)
Supplement: Table S1 — Human brain sections derived from patients with inflammatory injuries analyzed for binucleated Purkinje neurons. Cerebella from adult human brains, all from patients clinically diagnosed with inflammations, were analyzed for Purkinje neurons containing two nuclei. According to published data and our own results obtained from mouse brains, the inflammatory lesion can increase the percentage of fused or recombined Purkinje neurons from 0.2% to 14% of the total population. Assuming similar quantities in the human brain, we should have observed between 700 and 50,000 binucleated Purkinje neurons in all cases combined. (DOCX) [file pbio.1001874.s005.docx]

**Table S1. Human brain sections derived from patients with inflammatory injuries analyzed for binucleated Purkinje neurons.**

| No | Age | Sex | Pathology | Microglia activation, leucocyte invasion | PKN analyzed |
| --- | --- | --- | --- | --- | --- |
| 1 | 64 | m | Aspergillus meningitis with metastatic brain abscess, arachnoid cyst | yes | 3253 |
| 2 | 50 | m | HIV-Enzephalopathy, Cryptococcus-Meningoencephalitis | yes | 2096 |
| 3 | 72 | f | Myelitis disseminata | yes | 1132 |
| 4 | 0 | f | Aspergillus Meningitis | yes | 1178 |
| 5 | 34 | m | Cryptoccocus- Meningitis , Cryptococcus- Encephalopathy | yes | 1392 |
| 6 | 89 | f | chronical active Encephalomyelitis disseminata, Opticusatrophy, Status cribrosus, microvascular disease | yes | 1420 |
| 7 | 25 | m | Toxoplasma Encephalopathy, AIDS | yes | 1520 |
| 8* | 63 | m | Sepsis | no | 67.000 |
| 9* | 29 | m | AIDS, Hepatitis C | no | 60.000 |
| 10* | 50 | m | Alcohol abuse, depression | no | 115.000 |
| 11* | 57 | m | Alcohol abuse, liver cirrhosis, Pancreatitis | no | 94.000 |

m, male; f, female; *previously published cases [[7](#_ENREF_7)] that were clinically diagnosed with inflammation.
