# Supplementary material for: Extracellular Vesicle-Mediated Transfer of Genetic Information between the Hematopoietic System and the Brain in Response to Inflammation
Source: PLoS Biol. 2014 Jun 3;12(6):e1001874. doi: 10.1371/journal.pbio.1001874 (PMC4043485; doi:10.1371/journal.pbio.1001874)
Supplement: Table S2 — miRNAs detectable in recombined and nonrecombined Purkinje neurons. miRNAs identified by qPCR array analysis with a Cp<36 from material isolated from microdissected Purkinje neurons based on the presence or absence of X-Gal staining. (DOCX) [file pbio.1001874.s006.docx]

**Table S2: Assessment of RNA quality obtained by laser microdissection**

**
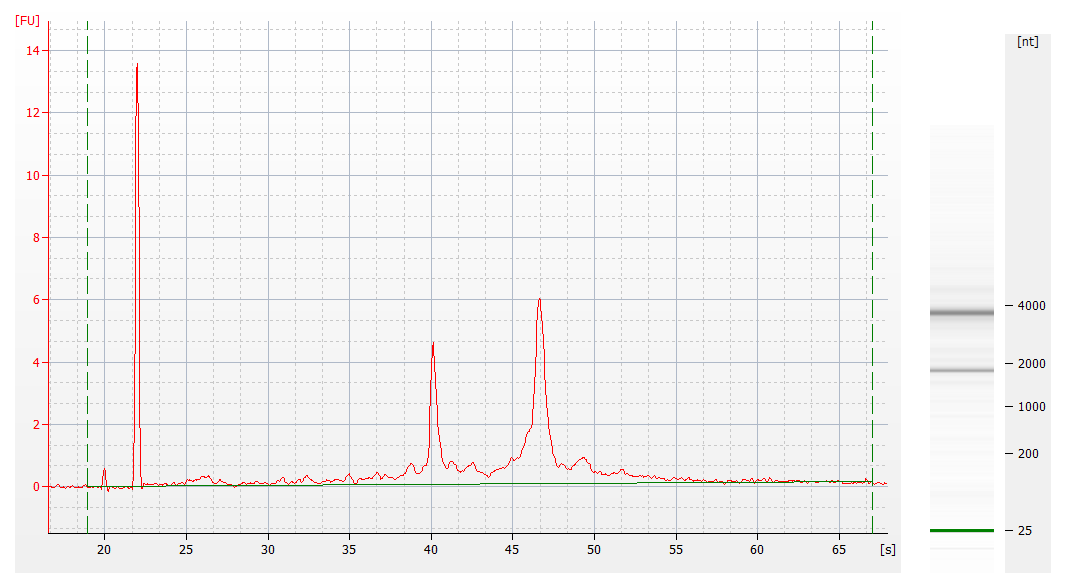
**

(18S:28S ration RIN: 8.2-8.8)

**Detection of target genes with SYBRGreen (PC – Purkinje cells, GCL – granule cell layer)**

| mRNA | Official full name (NCBI) | Primer (5' -> 3') | Size  (bp) | PC  (Cp) | GCL  (Cp) |
| --- | --- | --- | --- | --- | --- |
| *Gapdh* | glyceraldehyde-3-phosphate dehydrogenase | FP:ACAATGAATACGGCTACAG  RP:GGTCCAGGGTTTCTTACT | 78 | 30.07 | 28.82 |
| *Actb* | actin, beta | FP:GAAGATCAAGATCATTGCTCCT  RP:TGGAAGGTGGACAGTGAG | 84 | 30.71 | 29.23 |
| *Pgk1* | phosphoglycerate kinase 1 | FP:GTGATGAGGGTGGACTT  RP:TGGAACAGCAGCCTTGAT | 79 | 31.07 | 31.11 |
| *Ppia* | peptidylprolyl isomerase A | FP:AAGACTGAATGGCTGGAT  RP:ATGGCTTCCACAATGTTCA | 75 | 32.44 | 30.10 |
| *Calb2* | calbindin 2 | FP:AGTTCAATGCCATCTTCA  RP:TTCATCTCCTTCTTGTTCTT | 112 | > 35 | 31.09 |
| *Rbfox3* | RNA binding protein, fox-1 homolog (C. elegans) 3 | FP:TGAGATTTATGGAGGCTAT  RP:ATAACTGTCACTGTAGGC | 79 | > 35 | 29.52 |
| *Gfap* | glial fibrillary acidic protein | FP:AACCTGGCTGCGTATAGA  RP:CGAACTTCCTCCTCATAGAT | 125 | > 35 | > 35 |
| *Cspg4* | chondroitin sulfate proteoglycan 4 | FP:AGTTTACGCTCACCACTC  RP:AAATATGTCCACGTAGATAAAGTT | 89 | > 35 | > 35 |
| *Eno2* | enolase 2, gamma neuronal | FP:AAGATGGTGATCGGTATG  RP:TGATGTATCGGGAAGGAT | 100 | 32.55 | 30.93 |
| *Gap43* | growth associated protein 43 | FP:AGGAGAAAGACGCTGTAG  RP:AGTTCAGGCATGTTCTTG | 98 | > 35 | 31.79 |

**Detection of target genes with TaqMan Gene Expression Assays**

| mRNA | Official full name (NCBI) | TaqMan (ABI) | Size  (bp) | Cp  (PC) | Cp  (GCL) |
| --- | --- | --- | --- | --- | --- |
| *Gapdh* | glyceraldehyde-3-phosphate dehydrogenase | Mm99999915 | 107 | 34.09 | 32.16 |
| *Pcp2* | Purkinje cell protein 2 (L7) | Mm00435514_m1 | 59 | 32.60 | > 35 |
