# Supplementary material for: Extracellular Vesicle-Mediated Transfer of Genetic Information between the Hematopoietic System and the Brain in Response to Inflammation
Source: PLoS Biol. 2014 Jun 3;12(6):e1001874. doi: 10.1371/journal.pbio.1001874 (PMC4043485; doi:10.1371/journal.pbio.1001874)
Supplement: Table S3 — List of miRNAs associated with serum exosomes. List of all miRNAs identified by qPCR array analysis with a Cp<36 contained in the exosomal fraction (2–7) of blood sera from mice with or without an inflammation. (PDF) [file pbio.1001874.s007.pdf]

Table S3 miRNAs Cp<36 in LCM Purkinje neurons

Non-recombined Purkinje neurons

#397

miRNA	Cp
mmu-miR-669b	30,09
mmu-miR-30c	31,46
mmu-miR-9	31,77
mmu-miR-124	31,83
mmu-miR-26a	32,15
mmu-miR-883b-5p	32,22
mmu-miR-125a-5p	32,79
mmu-miR-30b	32,88
mmu-miR-125b-5p	32,91
mmu-miR-434-3p	33,82
mmu-let-7b	34,1
mmu-miR-126-3p	34,28
mmu-miR-328	34,45
mmu-miR-99b	34,46
mmu-miR-204	34,73
mmu-miR-181a	34,78
mmu-miR-127	34,87
mmu-miR-320	34,96
mmu-miR-342-3p	35,17
mmu-miR-195	35,27
rno-miR-340-3p	35,5
mmu-miR-24	35,53
mmu-miR-760-3p	35,53
mmu-miR-27a	35,63
mmu-miR-138	35,69
mmu-miR-206	35,71
mmu-miR-27b	35,85
mmu-let-7c	35,93

#304

miRNA	Cp
mmu-miR-124	29,13
mmu-miR-9	29,64
mmu-miR-30c	29,69
mmu-miR-26a	30,12
mmu-miR-125b-5p	30,15
mmu-miR-125a-5p	31,05
mmu-miR-30b	31,12
mmu-miR-669b	31,2
mmu-miR-138	31,76
mmu-miR-434-3p	32,02
mmu-let-7b	32,19
mmu-miR-126-3p	32,32
mmu-miR-204	32,62
mmu-miR-328	32,75
mmu-miR-99b	33
mmu-miR-195	33,37
mmu-miR-181a	33,55
mmu-miR-27b	33,65
mmu-miR-24	33,65
mmu-miR-342-3p	33,74
mmu-miR-127	33,9
mmu-miR-708	33,92
mmu-miR-320	33,94
mmu-miR-27a	34,09
mmu-let-7c	34,1
mmu-let-7g	34,34
mmu-miR-760-3p	34,43
mmu-miR-23a	34,57
mmu-let-7i	34,58
mmu-miR-30a	34,67
mmu-miR-154	34,72
mmu-miR-206	34,74
mmu-miR-21	34,75
mmu-miR-30d	34,79
mmu-miR-29a	34,79
mmu-miR-434-5p	34,88
mmu-miR-92b	34,96
mmu-miR-9*	34,97
mmu-miR-101a	34,99
mmu-miR-301a	35,06
mmu-miR-218	35,18
mmu-miR-99a	35,26
mmu-miR-101b	35,3
rno-miR-290	35,46
mmu-let-7e	35,48
mmu-miR-29c	35,53
mmu-miR-297b-3p	35,53
mmu-miR-30e	35,6
mmu-miR-324-5p	35,64
mmu-miR-149	35,64
mmu-miR-411	35,72
mmu-let-7a	35,75
mmu-miR-133b	35,83

Recombined Purkinje neurons

#397

miRNA	Cp
mmu-miR-582-3p	30,62
mmu-miR-26a	31,70
mmu-miR-30c	31,78
mmu-miR-345-3p	32,00
mmu-miR-124	32,41
mmu-miR-30b	32,46
mmu-miR-351	32,52
mmu-miR-16	32,63
mmu-miR-9	32,75
mmu-miR-125a-5p	33,19
mmu-miR-126-3p	33,26
mmu-let-7b	33,31
mmu-miR-434-3p	33,51
mmu-miR-204	33,54
mmu-miR-883b-5p	34,24
mmu-miR-99b	34,51
mmu-miR-490-3p	34,52
mmu-miR-23b	34,82
mmu-miR-196a-2*	34,89
mmu-miR-574-3p	34,95
mmu-miR-138	35,04
mmu-miR-190	35,05
mmu-miR-297c	35,08
mmu-miR-320	35,18
mmu-miR-328	35,23
mmu-miR-125b-5p	35,30
mmu-miR-24	35,35
mmu-miR-132	35,36
mmu-miR-342-3p	35,36
mmu-miR-27b	35,39
mmu-miR-212-3p	35,41
mmu-miR-154	35,45
mmu-miR-23a	35,48
mmu-miR-484	35,63
mmu-miR-27a	35,65
mmu-miR-127	35,67
mmu-miR-21	35,71
mmu-miR-486	35,72
mmu-miR-181a	35,73
mmu-miR-92b	35,78
mmu-miR-206	35,81
mmu-miR-301a	35,98

#304

miRNA	Cp
mmu-miR-669b	27,72
mmu-miR-124	30,19
mmu-miR-30c	30,20
mmu-miR-26a	30,30
mmu-miR-9	30,58
mmu-miR-883b-5p	31,19
mmu-miR-125b-5p	31,70
mmu-miR-30b	31,93
mmu-miR-125a-5p	32,13
mmu-let-7b	32,52
mmu-miR-126-3p	32,67
mmu-miR-582-5p	32,82
mmu-miR-204	32,89
mmu-miR-434-3p	33,08
mmu-miR-138	33,43
mmu-miR-16	33,60
mmu-miR-328	33,65
mmu-miR-342-3p	34,04
mmu-miR-297a	34,06
mmu-miR-27a	34,12
mmu-miR-668	34,23
mmu-miR-27b	34,27
mmu-miR-574-3p	34,33
mmu-miR-223	34,49
mmu-miR-127	34,52
mmu-miR-134	34,55
mmu-miR-669a-5p	34,72
mmu-miR-758	34,87
mmu-miR-29a	34,97
mmu-let-7g	35,01
rno-miR-223	35,02
mmu-miR-24	35,17
mmu-miR-222	35,19
mmu-miR-409-3p	35,29
mmu-miR-484	35,29
mmu-miR-466d-5p	35,31
mmu-miR-150	35,36
mmu-miR-191	35,45
mmu-let-7a-1*	35,52
mmu-miR-683	35,59
mmu-miR-877	35,60
rno-miR-290	35,65
mmu-let-7i	35,77
mmu-miR-149	35,81
mmu-miR-101b	35,83
mmu-miR-324-5p	35,83
mmu-miR-466d-5p	35,84
mmu-miR-214	35,85
mmu-miR-541	35,86
mmu-miR-412-3p	35,86
mmu-miR-20a	35,87
mmu-miR-218	35,97
mmu-miR-145	35,97
