# Supplementary material for: Extracellular Vesicle-Mediated Transfer of Genetic Information between the Hematopoietic System and the Brain in Response to Inflammation
Source: PLoS Biol. 2014 Jun 3;12(6):e1001874. doi: 10.1371/journal.pbio.1001874 (PMC4043485; doi:10.1371/journal.pbio.1001874)
Supplement: Table S4 — Assessment of RNA quality obtained by LCM. Estimating RNA fragmentation and specificity by testing for the expression of genes that are specific for Purkinje or other types of neurons as well as glial cells. Expression levels of Cp>35 were considered to be not expressed. (PDF) [file pbio.1001874.s008.pdf]

Table S4 miRNAs Cp<36 in serum exosomes

Exiqon	Cp peritonitis	Exiqon	Cp control
mmu-miR-451	24,28	mmu-miR-451	24,8
mmu-miR-144	25,74	mmu-miR-144	26,23
mmu-miR-142-3p	26,60	mmu-miR-142-3p	27,7
mmu-miR-16	27,36	mmu-miR-16	27,8
mmu-miR-21	27,39	mmu-miR-21	28,09
mmu-miR-223	28,48	mmu-miR-19b	29,16
mmu-miR-20a	28,95	mmu-miR-93	29,27
rno-miR-223	29,01	mmu-miR-223	29,29
mmu-miR-93	29,01	mmu-miR-486	29,31
mmu-miR-19b	29,01	mmu-miR-20a	29,34
mmu-miR-15b	29,05	mmu-miR-669b	29,41
mmu-miR-486	29,07	mmu-miR-15b	29,48
mmu-miR-126-3p	29,13	mmu-miR-26a	29,48
mmu-miR-150	29,16	mmu-miR-126-3p	29,67
mmu-miR-30c	29,20	mmu-miR-24	29,67
mmu-miR-23a	29,32	mmu-miR-23a	29,79
mmu-miR-15a	29,33	mmu-miR-15a	29,8
mmu-miR-26a	29,46	mmu-miR-30c	29,83
mmu-miR-24	29,57	rno-miR-223	29,9
mmu-let-7g	29,82	mmu-let-7g	29,96
mmu-miR-142-5p	29,99	mmu-miR-30b	30,19
mmu-miR-103	30,05	mmu-miR-103	30,25
mmu-miR-30b	30,10	mmu-let-7b	30,49
mmu-miR-191	30,24	mmu-miR-150	30,78
mmu-let-7i	30,45	mmu-miR-142-5p	30,9
mmu-miR-29a	30,51	mmu-miR-191	31,05
mmu-miR-222	30,65	mmu-miR-582-3p	31,05
mmu-let-7b	30,67	mmu-let-7i	31,18
mmu-miR-582-3p	30,81	mmu-miR-29a	31,27
mmu-let-7d	31,06	mmu-let-7d	31,35
mmu-miR-25	31,07	mmu-miR-27a	31,37
mmu-miR-27a	31,15	mmu-miR-23b	31,38
rno-miR-214	31,19	rno-miR-214	31,67
mmu-miR-328	31,25	mmu-miR-25	31,69
mmu-miR-23b	31,34	mmu-miR-328	31,72
mmu-miR-29c	31,40	mmu-miR-222	31,76
mmu-miR-320	31,44	mmu-miR-29c	31,8
mmu-miR-130a	31,47	mmu-miR-107	31,81
mmu-miR-128	31,51	mmu-let-7a	31,92
mmu-miR-760-3p	31,54	mmu-miR-31	31,93
mmu-let-7a	31,54	mmu-miR-320	31,96
mmu-miR-107	31,56	mmu-miR-128	31,97
mmu-miR-484	31,59	mmu-miR-101a	32,02
mmu-miR-101b	31,62	mmu-miR-125a-5p	32,03
mmu-let-7c	31,69	mmu-miR-322	32,06
mmu-miR-181a	31,82	mmu-miR-125b-5p	32,07
mmu-miR-101a	31,84	mmu-miR-181a	32,08
mmu-miR-125b-5p	31,91	mmu-miR-99a	32,15
mmu-miR-221	31,96	mmu-miR-484	32,19
mmu-miR-99a	31,97	mmu-let-7c	32,25
mmu-miR-151-5p	31,98	mmu-miR-26b	32,43
mmu-miR-27b	31,99	rno-miR-143	32,46
mmu-miR-322	32,03	mmu-miR-221	32,55
mmu-miR-342-3p	32,06	mmu-miR-101b	32,63
mmu-miR-425	32,23	mmu-miR-130a	32,68
mmu-miR-423-3p	32,30	mmu-miR-27b	32,69
mmu-miR-26b	32,33	mmu-miR-574-3p	32,72
mmu-miR-22	32,36	mmu-miR-151-5p	32,74
mmu-miR-29b	32,42	mmu-miR-423-3p	32,74
mmu-miR-32	32,47	mmu-miR-342-3p	32,75
mmu-miR-297a	32,50	mmu-miR-350	32,76
mmu-miR-350	32,53	mmu-miR-503	32,85
rno-miR-143	32,54	mmu-miR-29b	32,87
mmu-miR-106b	32,55	mmu-miR-425	32,88
mmu-miR-574-3p	32,68	mmu-miR-22	32,92
mmu-miR-186	32,78	mmu-miR-106b	32,99
mmu-miR-125a-5p	32,82	mmu-miR-1	33,2

mmu-miR-351	32,99
mmu-miR-669b	32,99
mmu-miR-17	33,02
mmu-miR-345-3p	33,03
mmu-miR-185	33,09
rno-miR-338	33,24
mmu-miR-147	33,27
mmu-miR-106a	33,28
mmu-miR-652	33,28
mmu-miR-99b	33,35
mmu-miR-758	33,36
mmu-miR-140	33,38
mmu-miR-148b	33,48
mmu-miR-1	33,48
mmu-miR-192	33,49
mmu-let-7f	33,49
mmu-miR-374	33,57
mmu-miR-19a	33,65
mmu-miR-423-5p	33,69
mmu-miR-503	33,69
mmu-miR-139-5p	33,71
mmu-miR-378	33,78
mmu-miR-30a	33,78
mmu-miR-30d	33,87
mmu-miR-143	34,00
mmu-miR-467a	34,01
mmu-let-7e	34,03
mmu-miR-301a	34,05
mmu-miR-205	34,07
mmu-miR-181d	34,14
mmu-miR-133b	34,17
mmu-miR-199a-3p	34,19
mmu-miR-326	34,20
mmu-miR-33	34,21
mmu-miR-148a	34,21
mmu-miR-210	34,26
mmu-miR-134	34,27
mmu-miR-199a-5p	34,28
mmu-miR-181c	34,31
mmu-miR-124	34,33
mmu-miR-151-3p	34,38
mmu-miR-133a	34,38
rno-miR-381	34,39
mmu-miR-31	34,40
mmu-miR-490-3p	34,45
mmu-miR-421	34,46
mmu-miR-18a	34,46
mmu-miR-466d-3p	34,49
mmu-miR-296-5p	34,51
mmu-miR-30e	34,52
mmu-miR-152	34,62
mmu-miR-497	34,64
mmu-miR-195	34,69
mmu-miR-200c	34,75
mmu-miR-338-3p	34,77
mmu-miR-540-3p	34,84
mmu-miR-450a	34,84
mmu-miR-297c	35,12
mmu-miR-466a-3p	35,17
mmu-miR-219-5p	35,19
rno-miR-136	35,19
mmu-miR-100	35,23
mmu-miR-672	35,26
mmu-miR-339-5p	35,27
mmu-miR-362-3p	35,27
mmu-miR-495	35,30
mmu-miR-874	35,35
mmu-miR-676	35,35
mmu-miR-28	35,37
mmu-miR-330	35,38

mmu-miR-297a	33,23
mmu-miR-652	33,33
mmu-miR-760-3p	33,4
mmu-miR-185	33,42
mmu-miR-351	33,43
mmu-miR-148b	33,43
mmu-let-7e	33,46
mmu-miR-133b	33,48
mmu-miR-301a	33,58
mmu-miR-186	33,66
mmu-miR-345-3p	33,74
mmu-miR-139-5p	33,75
mmu-miR-30a	33,76
mmu-miR-19a	33,77
mmu-miR-99b	33,82
mmu-miR-196a	33,84
mmu-miR-140	33,85
mmu-miR-423-5p	33,86
mmu-miR-17	34,02
mmu-miR-490-3p	34,02
mmu-miR-497	34,04
rno-miR-338	34,08
mmu-miR-374	34,08
mmu-let-7f	34,09
mmu-miR-32	34,15
mmu-miR-378	34,16
mmu-miR-152	34,16
mmu-miR-758	34,17
mmu-miR-30e	34,18
mmu-miR-467a	34,2
mmu-miR-324-5p	34,35
mmu-miR-365	34,35
mmu-miR-106a	34,36
mmu-miR-495	34,36
mmu-miR-199a-5p	34,42
mmu-miR-370	34,45
mmu-miR-326	34,47
mmu-miR-133a	34,47
mmu-miR-205	34,5
mmu-miR-148a	34,51
mmu-miR-143	34,65
mmu-miR-297b-3p	34,68
mmu-miR-181c	34,69
mmu-miR-134	34,71
mmu-miR-9	34,74
mmu-miR-466d-3p	34,76
mmu-miR-200c	34,79
rno-miR-345-5p	34,8
mmu-miR-296-5p	34,81
mmu-miR-210	34,82
rno-miR-200c	34,88
mmu-miR-30d	34,89
mmu-miR-195	34,94
mmu-miR-330	34,94
mmu-miR-409-3p	34,94
mmu-miR-122	34,95
mmu-miR-18a	35,13
mmu-miR-181d	35,25
mmu-miR-338-3p	35,25
mmu-miR-199a-3p	35,33
mmu-miR-192	35,45
mmu-miR-421	35,45
mmu-miR-33	35,57
mmu-miR-151-3p	35,89

mmu-miR-325	35,50
mmu-miR-331-3p	35,50
mmu-miR-362-5p	35,51
mmu-miR-194	35,53
mmu-miR-500	35,54
mmu-miR-196a-2*	35,55
mmu-miR-345-5p	35,56
mmu-miR-34a	35,66
mmu-miR-132	35,67
mmu-miR-146a	35,68
mmu-miR-434-3p	35,70
mmu-miR-181b	35,70
mmu-miR-324-3p	35,70
mmu-miR-145	35,72
mmu-miR-297b-3p	35,72
mmu-miR-30c-2*	35,90
mmu-miR-324-5p	35,92
mmu-miR-139-3p	35,97
