# Supplementary material for: Extracellular Vesicle-Mediated Transfer of Genetic Information between the Hematopoietic System and the Brain in Response to Inflammation
Source: PLoS Biol. 2014 Jun 3;12(6):e1001874. doi: 10.1371/journal.pbio.1001874 (PMC4043485; doi:10.1371/journal.pbio.1001874)
Supplement: Table S5 — Comparison of microdissected Purkinje neuron miRNAs with published miRNA profiles. Comparison of miRNAs detected in microdissected Purkinje neurons with miRNAs in three libraries (PKN 1–3, >10 pm reads) obtained from healthy animals in He et al. [31]. The total number of identified miRNAs contained in each library is given in brackets. (DOCX) [file pbio.1001874.s009.docx]

**Table S5. Comparison with published Purkinje neuron miRNA profiles.**

|  | **PKN1 (460)** | **PKN2 (445)** | **PKN3 (448)** |
| --- | --- | --- | --- |
| **#397 non-recombined (27)** | mmu-miR-883b-5p;  mmu-miR-340-3p | mmu-miR-883b-5p,  rno-miR-340-3p | mmu-miR-883b-5p  rno-miR-340-3p |
| **#304 non-recombined (53)** | rno-miR-290 | rno-miR-290  mmu-miR-297b-3p, | rno-miR-290  mmu-miR-297b-3p |
| **#397 recombined (41)** | mmu-miR-883b-5p  mmu-miR-196a-2*, mmu-miR-351,  mmu-miR-490-3p,  mmu-miR-574-3p | mmu-miR-883b-5p  mmu-miR-196a-2*, mmu-miR-490-3p,  mmu-miR-574-3p | mmu-miR-883b-5p  mmu-miR-196a-2*, mmu-miR-351,  mmu-miR-490-3p,  mmu-miR-574-3p,  mmu-miR-582-3p |
| **#304 recombined (52)** | mmu-miR-883b-5p,  rno-miR-290  mmu-miR-214,  mmu-miR-223,  mmu-miR-574-3p,  mmu-miR-683,  rno-miR-223 | mmu-miR-883b-5p,  rno-miR-290  mmu-miR-214,  mmu-miR-574-3p,  mmu-miR-683,  rno-miR-223 | mmu-miR-883b-5p,  rno-miR-290  mmu-miR-145,  mmu-miR-214,  mmu-miR-223,  mmu-miR-297a,  mmu-miR-574-3p,  mmu-miR-683,  rno-miR-223 |
