# Supplementary material for: Correction: Extracellular Vesicle-Mediated Transfer of Genetic Information between the Hematopoietic System and the Brain in Response to Inflammation
Source: PLoS Biol. 2018 Mar 12;16(3):e1002623. doi: 10.1371/journal.pbio.1002623 (PMC5846718; doi:10.1371/journal.pbio.1002623)
Supplement: S1 File — (PDF) [file pbio.1002623.s001.PDF]

OPEN ACCESS Freely available online

PLOS | BIOLOGY

Extracellular Vesicle-Mediated Transfer of Genetic Information between the Hematopoietic System and the Brain in Response to Inflammation

Kirsten Ridder[1,2], Sascha Keller[3], Maria Dams[1,2], Anne-Kathleen Rupp[3], Jessica Schlaudraff[4], Domenico Del Turco[4], Julia Starmann[2,5], Jadranka Macas[1,2], Darja Karpova[6], Kavi Devraj[1], Candan Depboylu[7], Britta Landfried[1], Bernd Arnold[8], Karl H. Plate[1,2], Günter Höglinger[7,9], Holger Sültmann[2,5], Peter Altevogt[3], Stefan Momma[1,2]*

1 Institute of Neurology (Edinger Institute), Frankfurt University Medical School Frankfurt, Frankfurt, Germany, **2** German Cancer Consortium (DKTK), German Cancer Research Center (DKFZ), Heidelberg, Germany, **3** Tumor Immunology Program, German Cancer Research Center (DKFZ), Heidelberg, Germany, **4** Institute of Clinical Neuroanatomy, Neuroscience Center, Frankfurt University Medical School Frankfurt, Frankfurt, Germany, **5** Unit Cancer Genome Research, German Cancer Research Center (DKFZ) and National Center for Tumor Diseases, Heidelberg, Germany, **6** German Red Cross Blood Service Baden-Württemberg-Hessen and Institute for Transfusion Medicine and Immunohematology, Frankfurt University Medical School, Frankfurt, Germany, **7** Department of Neurology, Philipps University Marburg, Marburg, Germany, **8** Division Molecular Immunology, German Cancer Research Center (DKFZ), Heidelberg, Germany, **9** Department for Translational Neurodegeneration, German Center for Neurodegenerative Diseases e.V. (DZNE), Technical University Munich (TUM), Munich, Germany

Abstract

Mechanisms behind how the immune system signals to the brain in response to systemic inflammation are not fully understood. Transgenic mice expressing Cre recombinase specifically in the hematopoietic lineage in a Cre reporter background display recombination and marker gene expression in Purkinje neurons. Here we show that reportergene expression in neurons is caused by intercellular transfer of functional Cre recombinase messenger RNA from immune cells into neurons in the absence of cell fusion. *In vitro* purified secreted extracellular vesicles (EVs) from blood cells contain Cre mRNA, which induces recombination in neurons when injected into the brain. Although Cre-mediated recombination events in the brain occur very rarely in healthy animals, their number increases considerably in different injury models, particularly under inflammatory conditions, and extend beyond Purkinje neurons to other neuronal populations in cortex, hippocampus, and substantia nigra. Recombined Purkinje neurons differ in their miRNA profile from their nonrecombined counterparts, indicating physiological significance. These observations reveal the existence of a previously unrecognized mechanism to communicate RNA-based signals between the hematopoietic system and various organs, including the brain, in response to inflammation.

Citation: Ridder K, Keller S, Dams M, Rupp A-K, Schlaudraff J, et al. (2014) Extracellular Vesicle-Mediated Transfer of Genetic Information between the Hematopoietic System and the Brain in Response to Inflammation. PLoS Biol 12(6): e1001874. doi:10.1371/journal.pbio.1001874

Academic Editor: Ben A. Barres, Stanford University School of Medicine, United States of America

Received February 24, 2014; **Accepted** April 24, 2014; **Published** June 3, 2014

Copyright: © 2014 Ridder et al. This is an open-access article distributed under the terms of the Creative Commons Attribution License, which permits unrestricted use, distribution, and reproduction in any medium, provided the original author and source are credited.

Funding: This study was supported by the Edinger Foundation and the Deutsche Forschungsgemeinschaft (DFG, grant DFG MO 2211/1-1). The funders had no role in study design, data collection and analysis, decision to publish, or preparation of the manuscript.

Competing Interests: The authors have declared that no competing interests exist.

Abbreviations: BBB, blood–brain barrier; BMCs, bone marrow cells; CNS, central nervous system; ECL, entorhinal cortex lesion; ELISA, Enzyme Linked Immunosorbent Assay; EM, electron microscopy; EVs, extracellular vesicles; GFAP, glial fibrillary acidic protein; GFP, green fluorescent protein; LCM, laser capture microdissected; LLC2, Lewis lung carcinoma cells; LPS, lipopolysaccharide; MPP+, 1-methyl-4-phenylpyridinium; MPTP, 1-methyl-4-phenyl-1,2,3,6-tetrahydropyridine; PBS, phosphate buffered saline; PC, Purkinje cell; PCR, polymerase chain reaction; PFA, paraformaldehyde; RT, reverse-transcriptase; SN, substantia nigra; TH, tyrosine hydroxylase; VTA, ventral tegmental area.

* E-mail: stefan.momma@kgu.de

These authors contributed equally to this work.

Introduction

The influence of the immune system on the brain in the context of inflammation is highly relevant for a number of diseases, yet mechanisms for this interaction are not fully understood. The stereotypical response is the secretion of pro-inflammatory cytokines by immune cells. These peripheral cytokines in turn can have a direct effect on neural cells or activate brain inflammatory cytokine signaling, usually via microglia, the principle innate immune cells of the brain [1].

Recently, heterotypic cell fusion of hematopoietic cells with Purkinje neurons in the brain has been suggested as a conceptually different mechanism of response to inflammation. When transplanted into lethally irradiated mice, genetically labeled hematopoietic donor cells have been found to contribute to a number of host tissues including skeletal and cardiac myofibers, hepatocytes in the liver, intestinal crypt cells, and Purkinje neurons in the brain. Initially seen as evidence for an unexpected differentiation potential of hematopoietic stem cells, it was eventually demonstrated that the experimentally observed plasticity was largely attributable to cell fusion, rather than transdifferentiation [2–4].

Author Summary

Peripheral infections leading to an inflammatory response can initiate signaling from the hematopoietic system to various organs including the brain. The traditional view of this communication between blood and brain is that individual factors are released by immune cells that in turn bind to neuronal or nonneuronal target cells in the brain where they exert their effects. By using a genetic tracing system, we now show that extracellular vesicles, small membrane structures that can contain a multitude of different molecules, can transfer functional RNA directly from blood cells to neurons. Although this type of signaling is highly restricted in the healthy animal, inflammatory injuries increase both the frequency of transfer and the range of the neuronal target populations in the brain. By showing altered miRNA profiles in neurons receiving extracellular vesicle cargo, we predict a complex regulation of gene expression in neural cells in response to peripheral inflammation.

For the brain, fusion of hematopoietic cells has so far mainly been reported with Purkinje neurons. Although the number of fusion events is very low in the healthy animal, peripheral inflammation induces cell fusion events to increase by a factor of 10–100, giving the first indication that heterotypic fusion is regulated by a pathologic stimulus and may therefore be of biological significance [5]. We were interested in studying the contribution of hematopoietic cells to neural tissue without the accompanying confounding factors such as lethal irradiation, chemoablation, or parabiosis normally associated with replacing the host bone marrow. In contrast, to irreversibly label hematopoietic cells to follow their fate *in vivo*, we used transgenic mice expressing Cre recombinase under the hematopoietic-specific promoter vav [6] in a Cre reporter background. Although in our mouse model we could observe recombination events in the same tissues and at a frequency comparable to those observed in transplantation studies, we did not find any evidence for cell fusion in Purkinje neurons, marked by the absence of a second nucleus [7]. We now show that recombination in neural cells is caused by the intercellular transfer of Cre recombinase messenger RNA. More specifically, biochemical analysis demonstrates that Cre mRNA is contained in extracellular vesicles (EVs), including exosomes. These EVs are sufficient to induce recombination in neural cells after direct intracerebellar injection. Recombination events occur rarely and are restricted to very few Purkinje neurons in the healthy animal. However, when inducing a peripheral inflammation or an entorhinal cortex lesion (ECL), the number of recombined cells increases dramatically and extends to other neuronal cell populations. Importantly, recombined versus nonrecombined Purkinje neurons display differences in their miRNA profile several days after inflammation, indicating biologically significant changes. These observations reveal the existence of a previously unrecognized mechanism to communicate RNA-based signals between the hematopoietic system and various organs, including the brain, in response to inflammation.

Results

Expression of Cre Recombinase Specifically in the Hematopoietic Lineage Leads to Recombination Events in Purkinje Neurons Without Cell Fusion

To monitor the contribution of hematopoietic cells to other tissues without any of the confounding factors associated with host bone marrow replacement, we previously utilized transgenic mice expressing Cre recombinase specifically in the hematopoietic lineage under the Vav1 promoter [7]. For this study, we additionally included a mouse model expressing Cre under the hematopoietic- and endothelial-specific promoter Tie2 [8] to minimize the possibility of false positive results due to a leaky expression of Cre. Thus, in hematopoietic cells, marker gene expression is irreversibly induced (Figure 1A), allowing the tracing of hematopoietic contribution to any other tissue. Confocal analysis of different tissue sections of both Vav-iCre- and Tie2-Cre-GFP/LacZ mice showed GFP- or LacZ-positive cells in organs such as the liver, lung, and small intestine (Figure 1B–D) similar to observations made in animals transplanted with bone marrow from constitutively marker-gene-expressing cells. In the cerebellum, only Purkinje neurons could be observed expressing the marker gene (Figure 1E). However, all marker-gene–positive Purkinje neurons from both mouse lines contained only a single nucleus in line with our previous findings [7], suggesting a contribution of hematopoietic-to-neural cells independent of cell fusion (Figure 1F). Thus, in both our transgenic models we could make observations similar to what had been reported for chimeric animals but with no evidence of cell fusion in neurons.

Hematopoietic Cell-Derived Exosomes Contain Cre mRNA

The observation of Cre-dependent reporter gene expression without evidence of cell fusion events led us to consider alternative explanations for a transfer of Cre recombinase from hematopoietic cells to neurons. A growing body of evidence suggests a role for EVs in cell-to-cell communication [9]. Moreover, EVs can transfer functional mRNA between cells *in vitro* [10]. We therefore asked if reporter gene expression in nonhematopoietic cells was the result of an RNA-based transfer of Cre recombinase via EVs, leading to the translation of Cre RNA in target cells and nuclear recombination followed by irreversible reporter gene expression. Because low levels of recombination events could be detected in uninjured animals, we hypothesized that Cre messenger RNA should be detectable in the blood of mice with hematopoietic Cre recombinase expression. Peripheral blood was drawn from six Vav-iCre mice and processed according to a protocol for the isolation of secreted membrane vesicles [11]. In all vesicle isolation experiments, the pellet was treated with RNaseA to remove RNA that was not contained in vesicles. RNA was extracted from vesicle preparations and reverse transcribed, followed by nested primer RT-PCR. Cre recombinase cDNA could be detected in four out of six analyzed blood samples (Figure 2A), showing that Cre mRNA is present at low levels in blood plasma. To obtain larger amounts of secreted membrane vesicles for a more detailed analysis, we prepared *in vitro* cultures from pooled peripheral blood and bone marrow of Vav-iCre mice ($n = 6$ separate preparations from one to two mice each) and added lipopolysaccharide (LPS) at 200 ng/ml to stimulate an inflammatory reaction. Cell culture supernatants were collected after 3 d of stimulation, and membrane vesicle fractions enriched for exosomes were prepared via differential ultracentrifugation. The resulting supernatants and pellets were used for RNA extraction, reverse transcription, and subsequent detection of cDNA using PCR. Consistently, Cre recombinase cDNA could be detected in the vesicle pellet but not in the supernatant (Figure 2B) in all experiments, suggesting that Cre mRNA was not part of the soluble cell culture medium but was contained in physical structures amenable to separation by centrifugation. To directly show that Cre mRNA is contained in vesicles and does not sediment because of an association with protein complexes, we took vesicle pellets prepared as above and

treated them with RNaseA alone or in combination with detergent. In this way, vesicles would be lysed and the RNA exposed to RNase digestion. Indeed, in the samples treated with detergent and RNAse, we could no longer detect Cre mRNA in contrast to vesicle pellets that were treated with RNAse alone ($n=2$ independent sample preparations) (Figure 2C). Electron microscopic images from our vesicle preparations showed predominantly structures with sizes between 50 and 100 nm and a round or cup-shaped morphology typically associated with exosomes (Figure 2D) [12]. EVs are a heterogeneous population and their typology is not established with regard to their possible biological functions. However, the most prominent subclass are exosomes as defined by their size, protein marker load, and enrichment profile by differential density ultracentrifugation [9]. To specifically determine whether exosomes contain Cre mRNA, we fractionated the vesicle preparations using sucrose density gradient ultracentrifugation [13]. Exosomal identity was confirmed by Western blot analysis for the specific surface markers ADAM10 and CD9 (Figure 2E,F). Using antibodies against Cre recombinase, we could not detect Cre recombinase protein in any of the fractions (Figure 2G). We could also not detect any Cre protein by ELISA with a sensitivity of 0.1 ng/ml in total EV preparations of bone marrow and peripheral blood of a single mouse (unpublished data). Thus, our EV preparations do not contain Cre protein or only at exceedingly low concentrations. Cre recombinase RNA could always be detected by RT-PCR in all exosomal fractions (Figure 2H). Nonexosomal fractions varied with regard to the presence of Cre mRNA, ranging from its complete absence (Figure S1A) to individual subfractions being positive for Cre-derived cDNA ($n=5$ preparations) (Figure 2H). The latter observation indicated either trace amounts of exosomes in fractions negative for exosomal markers or Cre mRNA containing nonexosomal vesicles. Quantitative analysis of vesicle number and size revealed an increase in the share of larger membrane blebs or apoptotic vesicles in the *in vitro* preparations compared to blood plasma (Figure S1B). Our results demonstrate that Cre mRNA but not Cre recombinase protein is contained predominantly in exosomes and suggests that functional Cre

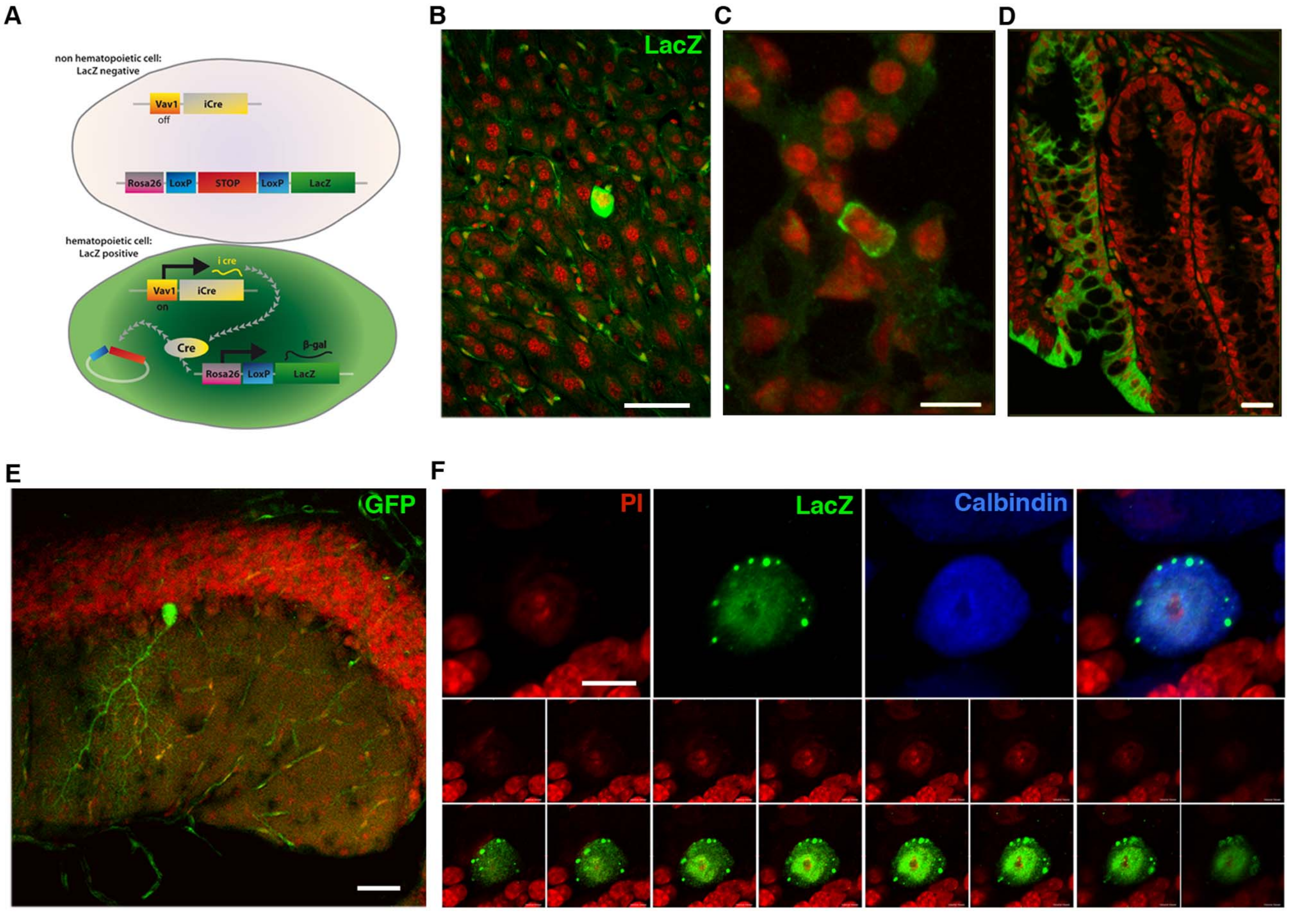

Figure 1. Noninvasive tracing of hematopoietic Cre recombinase activity. (A) Expressing Cre recombinase specifically in the hematopoietic lineage reveals the contribution of hematopoietic cells to nonhematopoietic tissues by irreversibly switching on reporter gene expression after excision of a floxed stop codon. β-galactosidase expression after recombination can be observed in multiple tissues such as liver (B), lung epithelia (C), and small intestine (D). In the intestine, the labeling of an entire crypt indicates recombination of an intestinal stem cell with concomitant inheritance of the marker to all progeny. (E) Overview of a cerebellar section showing a single recombined Purkinje neuron expressing GFP (green). (F) High magnification of a Purkinje neuron in serial section confocal analysis confirming absence of a second nucleus, excluding cell fusion. Scale bar, 50 µm (B), 20 µm (C), 50 µm (D), 50 µm (E), and 10 µm (F).
doi:10.1371/journal.pbio.1001874.g001

recombinase protein was generated from Cre RNA contained in vesicles rather than recombination activity being a result of the direct transfer of Cre protein.

Injection of Cre mRNA-Containing EV Preparations Is Sufficient to Induce Recombination in the Cerebellum

We wanted to test whether secreted Cre recombinase RNA-containing EVs are sufficient to induce recombination in Purkinje neurons *in vivo*. To this end, EVs enriched for the exosomal fraction were prepared from peripheral blood and bone marrow cultures from Vav-iCre mice as described above. These preparations were brought into the circulation of ROSA26-lacZ reporter mice intravenously (Figure 3A). Four days after injection, animals were transcardially perfused and analyzed for recombination events by X-Gal staining and immunohistochemistry in serial sections of the cerebellum. We could not observe recombination events in any of the brains of the animals analyzed ($n = 4$, unpublished data). These results suggested either that the amount of exosomes in our preparations was too low, leading to the quantitative absorption of vesicles *en route* to the brain, or that EVs could not cross the blood–brain barrier (BBB). To circumvent these problems and to test whether induction of reporter gene expression in Purkinje neurons by Cre mRNA-containing EVs is formally possible, we directly injected 1 µl vesicle preparations into the cerebella of ROSA-LacZ reporter mice ($n = 3$, two injections in a single hemisphere of each mouse; Figure 3A). Serial sections of the complete cerebellar hemisphere were analyzed for reporter gene expression 4 d after injection. This analysis identified Purkinje neurons expressing the reporter gene in all three injected animals with one to seven recombined Purkinje neurons in a single hemisphere (Figure 3B). Interestingly, we also observed other reporter gene-positive cell types with glial (Figure 3C) or microglial (Figure 3D) morphology in addition to Purkinje neurons in all injected animals. Hence, Cre mRNA-containing EV preparations are sufficient to induce recombination events in the brain, and the transfer of functional Cre mRNA by EVs appears not to be necessarily restricted to Purkinje neurons. Control brains from ROSA-LacZ mice that were injected with purified recombinant Cre recombinase protein (1 µl of 1 U/µl) ($n = 4$) or lysate prepared from Vav-iCre bone marrow cells (BMCs) ($n = 2$) did not display reporter gene expression in Purkinje neurons or any other cell types (Figure 3E).

Recombination Events in Purkinje Neurons Are Not the Result of an Endogenous Misexpression of Cre Recombinase

To formally rule out the possibility that the recombination events we observe were resulting from an unspecific expression of Cre, we performed additional control experiments. First, we established cerebellar slice cultures from Vav-iCre- or Tie2-Cre-ROSA-LacZ/GFP mice ($n = 4$). After 2 d in culture, we induced an injury by transversally cutting into the tissue with a blade, and 3 d later slices were analyzed. We could only detect occasional recombination events in the Purkinje cell layer and no recombination in the injured areas, indicating a lack of Cre activity in the absence of a hematopoietic system. Next, we generated blood chimeras by transplanting Vav-iCre-GFP bone marrow into lethally irradiated ROSA-LacZ reporter mice ($n = 6$). In this way, we could identify cells that received Cre recombinase from the donor population while controlling for the possibility of cell fusion through the constitutive expression of GFP in the donor cells. After 5 wk or 2 mo, respectively, we induced peritonitis and 4 d later the chimeric animals were killed (for an experimental scheme, see Figure 4A). We could observe LacZ-positive, GFP-negative cells in the livers of all animals ($n = 6$; Figure 4B). In the cerebellum, recombined cells became apparent only in the longer

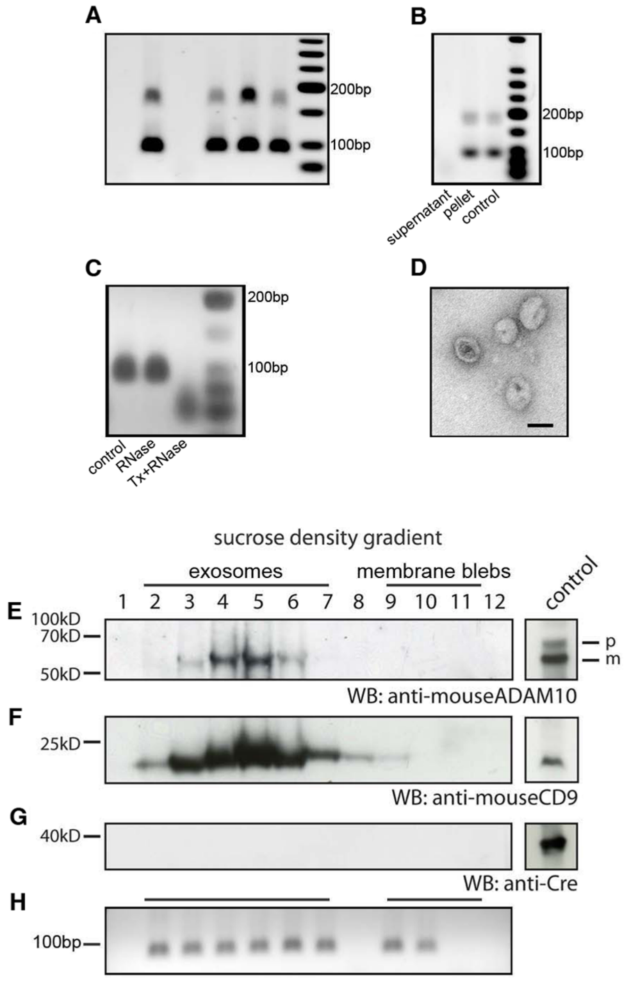

Figure 2. Cre mRNA is present in the blood plasma of Vav-iCre mice and contained in EVs including exosomes. (A) Cre mRNA can be detected in vesicle preparations enriched for exosomes from the blood plasma of Vav-iCre mice by RT-PCR. Each lane represents a result from an individual animal. For detection of Cre mRNA, nested primer PCR was used. The PCR product at 100 bp represents the signal after the second round of amplification. (B) Cre mRNA is localized in the pellet but not in the supernatant after ultracentrifugation of conditioned medium from primary Vav-iCre–positive hematopoietic cells after stimulation by LPS *in vitro*. Cre mRNA was resistant to RNaseA treatment in all experiments. (C) After treatment with Triton-X to lyse EVs in combination with RNaseA digestion, Cre mRNA is no longer detectable in contrast to RNaseA treatment alone. (D) Vesicular structures between 50 and 100 nm in size were visualized in electron micrographs from Vav-iCre hematopoietic-cell-derived vesicle preparations (scale bar, 50 nm). (E and F) Secreted membrane vesicle subspecies can be separated by density by sucrose gradient ultracentrifugation. Exosomal identity was confirmed by blotting against the specific protein markers ADAM10 and CD9 for all subfractions. (G) Cre protein could not be detected in any of the fractions. Positive controls for all antibodies are shown in boxes to the right. (H) Cre mRNA is present in the exosomal fractions 2–7. The nonexosomal vesicles fractions or apoptotic bodies are characterized by their variability of positive subfractions to complete absence of Cre mRNA. In this experiment, subfractions 9 and 10 are positive, whereas 8, 11, and 12 do not contain any Cre RNA.
doi:10.1371/journal.pbio.1001874.g002

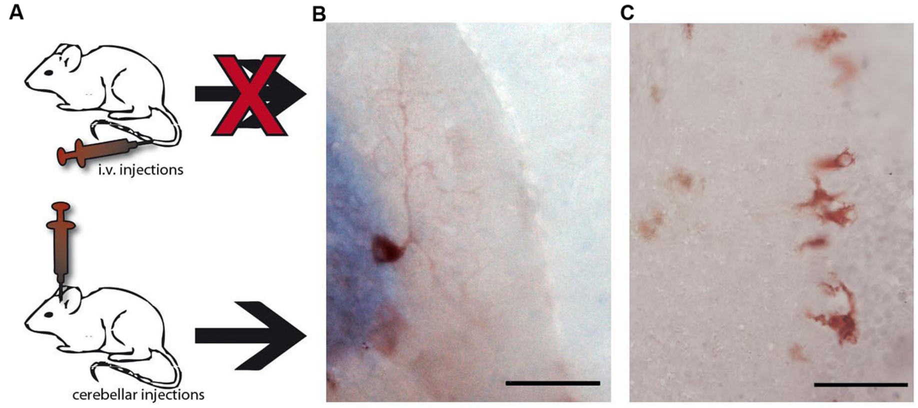

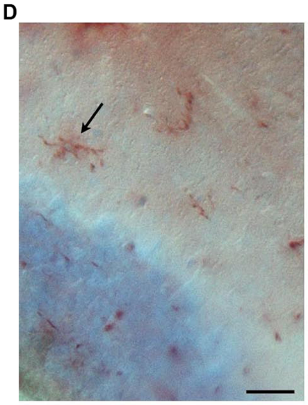

E

mouse no.	age (months)	sex	Recombination in Purkinje neurons (n)	Recombination in other cell types
1	12	m	1	Yes
2	12	m	7	Yes
3	11	f	5	Yes
4	9	f	0	No
5	4	m	0	No
6	12	m	0	No
7	16	m	0	No
8	10	f	0	No
9	14	m	0	No

Figure 3. EVs containing Cre mRNA are sufficient to induce recombination in Purkinje neurons after intracerebellar injection *in vivo*. (A) EV preparations enriched for exosomes prepared from the peripheral blood and bone marrow of Vav-iCre mice were brought into the circulation by tail vein injection or were directly injected into the cerebellum. Injection of Cre RNA-containing EVs into tail veins did not lead to recombination events in the brain ($n = 4$). (B) β-galactosidase–positive Purkinje neuron in the cerebellum of a reporter mouse 4 d after intracerebellar injection of EVs. (C) Other reporter-gene–positive cells with a shape and size reminiscent of glial cells in proximity to the Purkinje cell layer. (D) Reporter-gene–positive cells displaying a microglia-like morphology. (E) Quantification of reporter-gene–expressing Purkinje neurons after intracerebellar injection of vesicle preparations from Vav-iCre–positive peripheral blood. Control mice (shaded part) were injected with 1 µl purified Cre-recombinase protein at 1 U/µl (light grey) or lysate prepared from Vav-iCre bone marrow (dark grey) and never showed any recombined cells. Scale bar, 50 µm (B and C) and 25 µm (D).
doi:10.1371/journal.pbio.1001874.g003

surviving animals (2 mo, $n = 3$). We could identify β-galactosidase–positive, GFP-negative cells in the Purkinje layer (3.3±1.2 SD in one hemisphere) but also in the granular layer (5±1.7 SD in one hemisphere) as well as in cells associated with blood vessels (Figure 4C–E). In the latter two locations, we never observed recombined cells in the Vav-iCre/Tie2-Cre-GFP/LacZ mice, indicating physiological changes caused by the irradiation. To test whether we could still observe recombination events in chimeras without the confounding influence of lethal irradiation, we performed adoptive transfer experiments whereby 20 million spleen/lymph node cells from Vav-iCre-GFP mice were i.v. injected into ROSA-LacZ reporter mice ($n = 8$). Ten days after the transfer, animals were killed and their livers and brains analyzed. Although we could detect recombined, GFP-negative cells in the livers of four mice, we did not observe any recombination in the brain, probably due to the low and short-lived cell number present in the recipients (Figure 4A). Control animals for both types of experiments included ROSA-LacZ reporter mice as recipients injected with wild-type donor cells ($n = 4$). In sum, these experiments present formal evidence of a lateral transfer of Cre mRNA originating from blood cells, excluding fusion or endogenous misexpression.

Systemic and Central Nervous System (CNS) Inflammation Increases Recombination in Purkinje Neurons

Hematopoietic cells of the myeloid and lymphoid lineage have been shown to contribute to nonhematopoietic tissues by heterotypic cell fusion [14]. These fusion events are very rare, but their number increases substantially after peripheral inflammation [5], leading to speculation that heterotypic fusion and subsequent reprogramming of the hematopoietic nucleus represent a sort of rescue mechanism for damaged tissues [15]. Likewise, inflammatory stimuli may affect EVs signaling [16,17]. We induced a chronic inflammation by injecting Lewis lung carcinoma cells (LLC2) into the flanks of double transgenic mice, leading to the formation of a peripheral tumor after 12 d. For an acute inflammation, we induced peritonitis by intraperitoneal injection of thioglycolate broth and killed the animals 4 d after injection. In both models, we observed a significant increase in the number of recombined Purkinje neurons of up to three orders of magnitude (Figure 5A–C). To complement these models of a systemic inflammation with a pathology that directly affects neural tissue, we induced an ECL [18] in Vav-iCre-LacZ mice ($n = 3$), and 4 d postlesion, we observed a significant increase in the number of

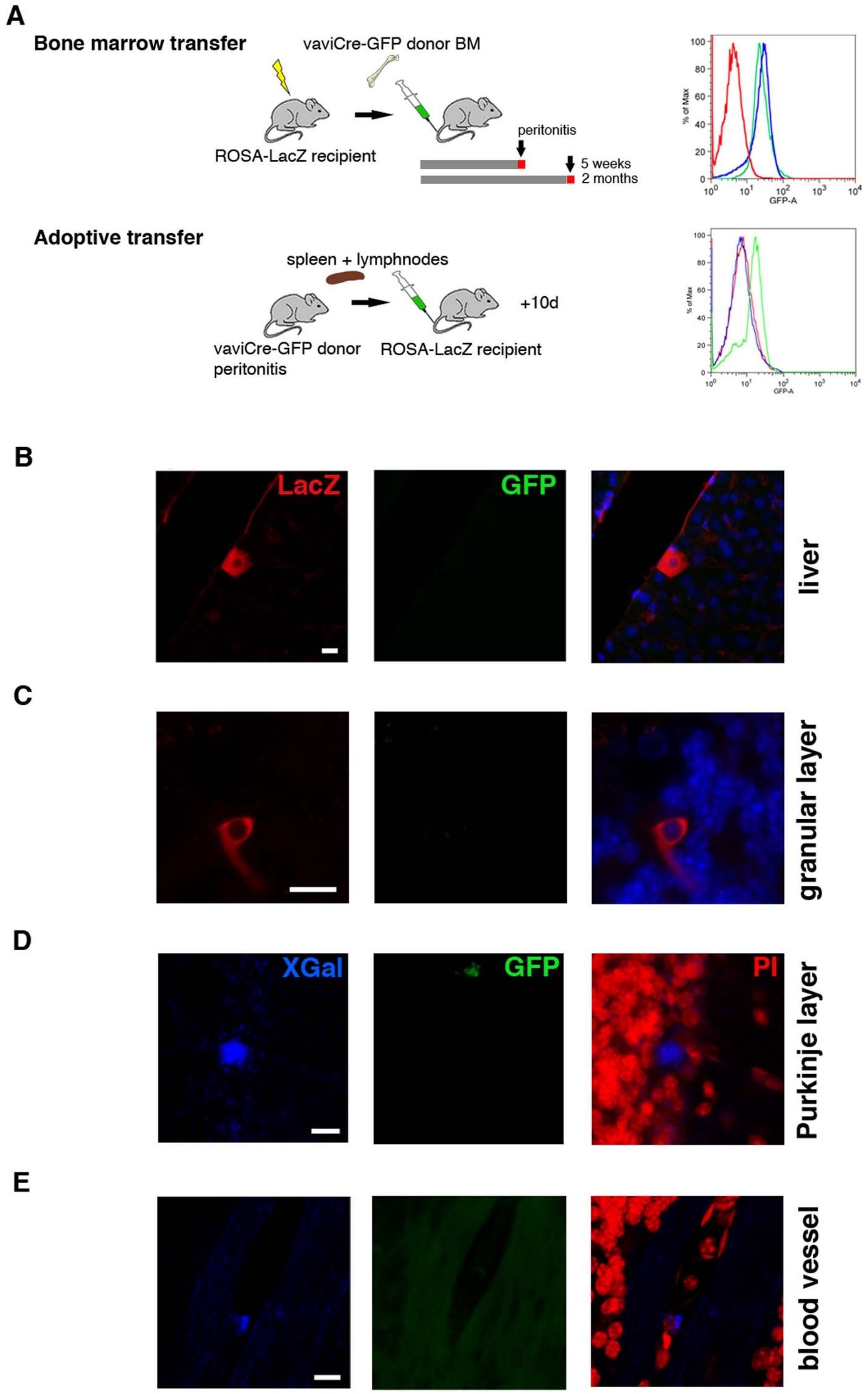

Figure 4. Transfer of Cre mRNA in blood chimeras. (A) Schematic drawings of the experimental strategies to test for recombination events in blood chimeras. Lethally irradiated ROSA26-LacZ mice receive BM cells from Vav-iCre-ROSA-GFP mice. The bone marrow of recipient mice was tested for engraftment by flow cytometry analysis of GFP expression (representative analysis in right panel; wild-type bone marrow, red line; Vav-iCre-ROSA-GFP donor bone marrow, green line; bone marrow of ROSA26-LacZ recipient mouse after engraftment, blue line). For adoptive transfer experiments, the same combination of transgenes was used with spleen and lymph node cells as donor organs. Representative flow cytometry analysis of GFP expression of donor (green line) compared to wild-type cells (red line). At the time of analysis, GFP-positive cells were undetectable in recipient spleens (blue line). Two months after bone marrow transplantation, recombined cells can be detected in the liver (B), granular cell layer (C), and Purkinje cell layer (D), as well as associated with blood vessels (E). None of the recombined LacZ-/X-Gal–positive cells were positive for GFP, excluding cell fusion. Scale bar, 10 µm (B–E).
doi:10.1371/journal.pbio.1001874.g004

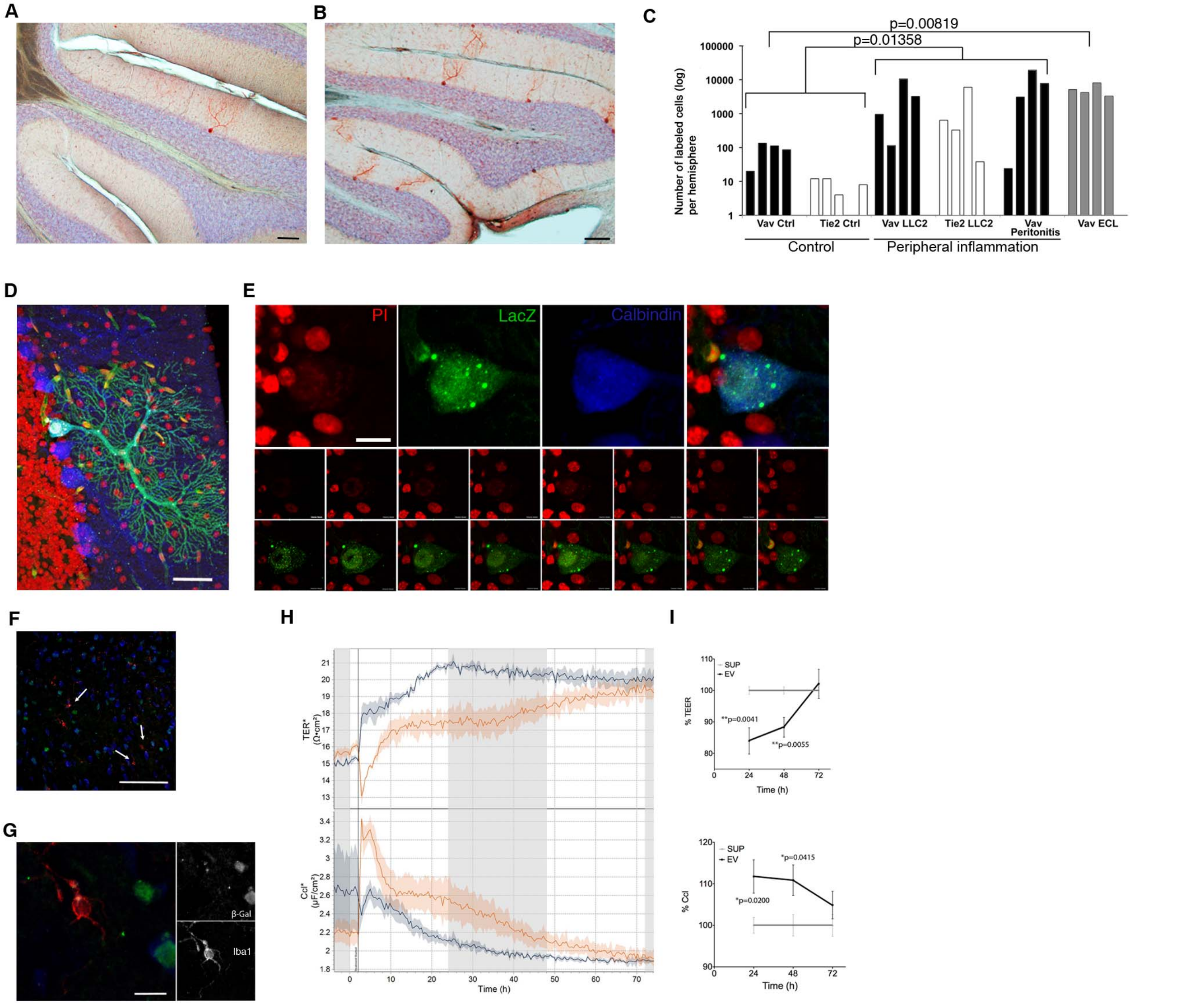

Figure 5. Peripheral inflammation increases the number of recombined Purkinje neurons. The number of recombined Purkinje neurons is low in healthy animals (A) but increases dramatically after peripheral inflammatory conditions (B). Inflammatory injuries were induced by subcutaneous injection of LLC2s or peritonitis. Animals were analyzed 12 d after injection when tumors were formed. Peritonitis was induced by a single i.p. injection of thioglycolate broth (1 ml in 3% PBS). Mice with peritonitis and ECL were analyzed 4 d after injection. (C) Filled bars represent results from Vav-iCre and empty bars from Tie2-Cre reporter mice. The *p* values were calculated by two-tailed *t* test for groups with unequal variance. (D and E) We did not observe any recombined Purkinje neurons that were binucleated in either transgenic mouse line after induction of an inflammation. (F and G) Microglia (white arrows) were always negative for the marker gene in healthy animals as well as after an inflammation. (H and I) Transendothelial electrical resistance (TEER, top panel) decreases and the corresponding capacitance (Ccl, bottom panel) of the bEnd5 endothelial monolayers increases significantly 24 h and 48 h after addition of bone-marrow-derived EVs compared to conditioned medium supernatant after ultracentrifugation. Vertical line at 0 h indicates media exchange. Scale bar, 100 µm (A and B), 50 µm (D and F), 10 µm (E), and 5 µm (G).
doi:10.1371/journal.pbio.1001874.g005

recombined Purkinje neurons (Figure 5C). Altogether, on the basis of a total population of approximately 150,000 Purkinje neurons per cerebellum, the relative quantity of recombined Purkinje neurons after inflammation thus averages 5.8% but goes as high as 26% in one individual case. Importantly, none of the recombined cells that we had analyzed in more detail were binucleated (Figure 5D,E), indicating that in our model systems injury is not sufficient to induce heterotypic cell fusion. The numbers of recombined Purkinje neurons for both Cre mouse lines were comparable to increases observed in heterologous transplantation models [5,14]. Of note, when screening a large number of sections of postmortem human cerebellar tissue from patients ($n = 12$) that suffered from severe inflammatory injuries, we could very rarely detect Purkinje neurons containing two nuclei (Figure S2, Table S1), consistent with observations made in our transgenic mouse models.

Microglia are the main immune cells of the brain and are able to secrete EVs [19]. Therefore, we examined whether this cell type could be a possible source of Cre RNA-containing EVs in our model. We first analyzed brains from early postnatal (P4–P8) double transgenic Vav-iCre/Tie2-Cre-LacZ mice—the peak time of microglia invasion to the brain. In line with recent findings that

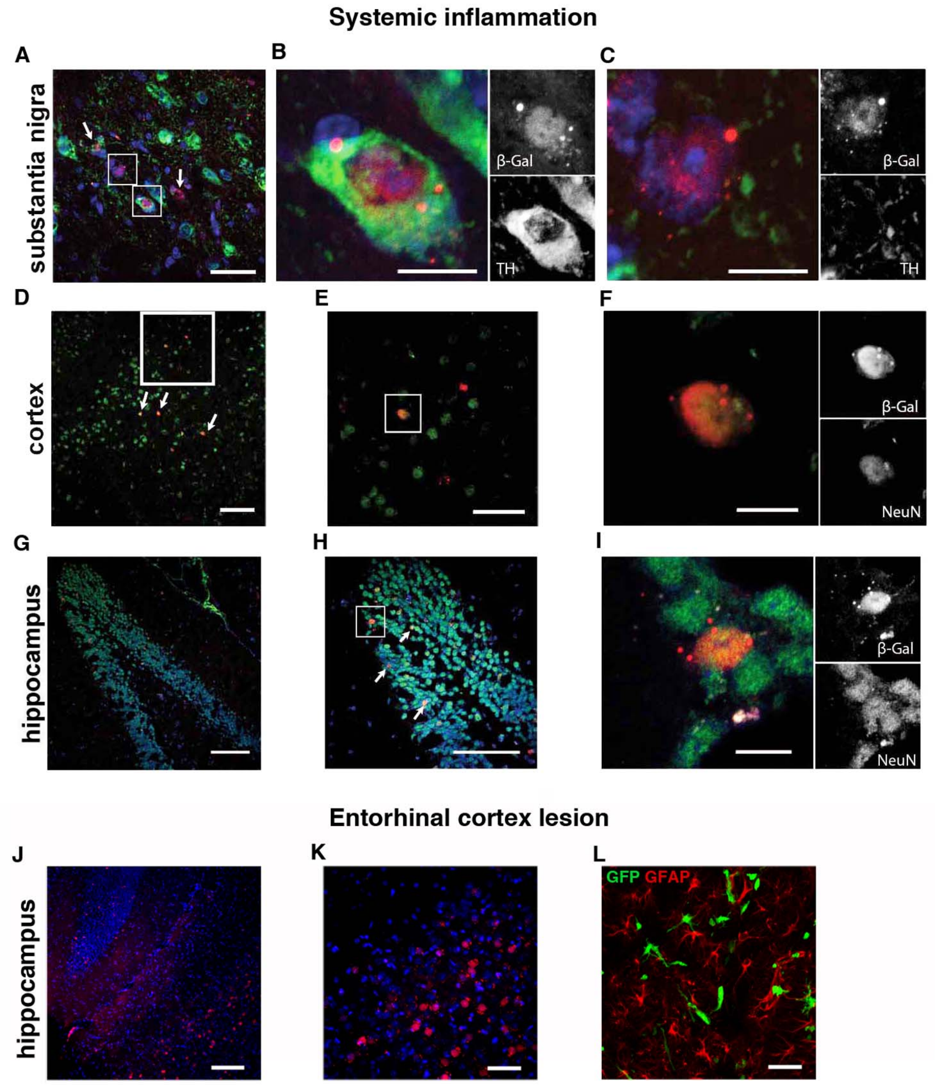

Figure 6. Reporter-gene–positive neurons can be found in multiple areas of the brain. (A and B) β-galactosidase–positive cells in the SN/VTA that can be TH-positive dopaminergic neurons as well as TH-negative cells with neuronal morphology (C). (D–F) β-galactosidase–positive cells in the cortex are also positive for NeuN. (G–I) Overview of the dentate gyrus in the hippocampus with recombined neurons in the granular cell layer that are positive for the neuronal marker NeuN. (J and K) Recombined neurons after ECL in hippocampal areas CA2 and CA3 and nonneuronal GFAP-negative recombined cells at the lesion site (L). Scale bar, 100 µm (D, G, H, and J), 50 µm (A, E, K, and L), and 10 µm (B, C, F, and I).
doi:10.1371/journal.pbio.1001874.g006

the origin of microglia precedes definitive hematopoiesis [20], none of the microglia were positive for the reporter gene (unpublished data). Equally, we did not observe microglia expressing the reporter gene either in the healthy adult brain or after inflammatory injuries (Figure 5F,G). Thus, in our model, Cre-mediated recombination activity in neurons is not induced by EVs of microglial origin.

EVs Targeting Neurons May Enter the Brain Via the Circulation

To gain insight into whether blood-derived EVs reach Purkinje neurons directly via the circulation or by entry of leukocytes into the brain and subsequent local release of EVs, we screened serial cerebellar sections from mice with peritonitis for CD45-LacZ double-positive cells ($n = 6$). We did not detect a single double-positive cell in the brain parenchyma, arguing against a transfer of EVs from leukocytes to neurons over a short distance. Next, we wanted to test whether EVs themselves may influence BBB properties in order to facilitate their passage into the brain. To this end, we measured changes in the electric resistance and capacitance over a monolayer of bEnd5 brain endothelium cells, a system that has been previously described as an adequate approximation of BBB properties *in vitro* [21]. EVs from three separate preparations from the supernatant of BMCs were added to the transwells (three separate measurements in quadruplicate). The supernatants of these EV preparations after the ultracentrifugation step served as negative controls. Addition of EVs, but not the supernatant, led to a decrease in the resistance and a concomitant increase in the capacitance at 24 h and 48 h but not any more at 72 h (Figure 5H,I). This suggests that EVs from the bone marrow are sufficient to make the BBB more permeable without lasting toxic effects.

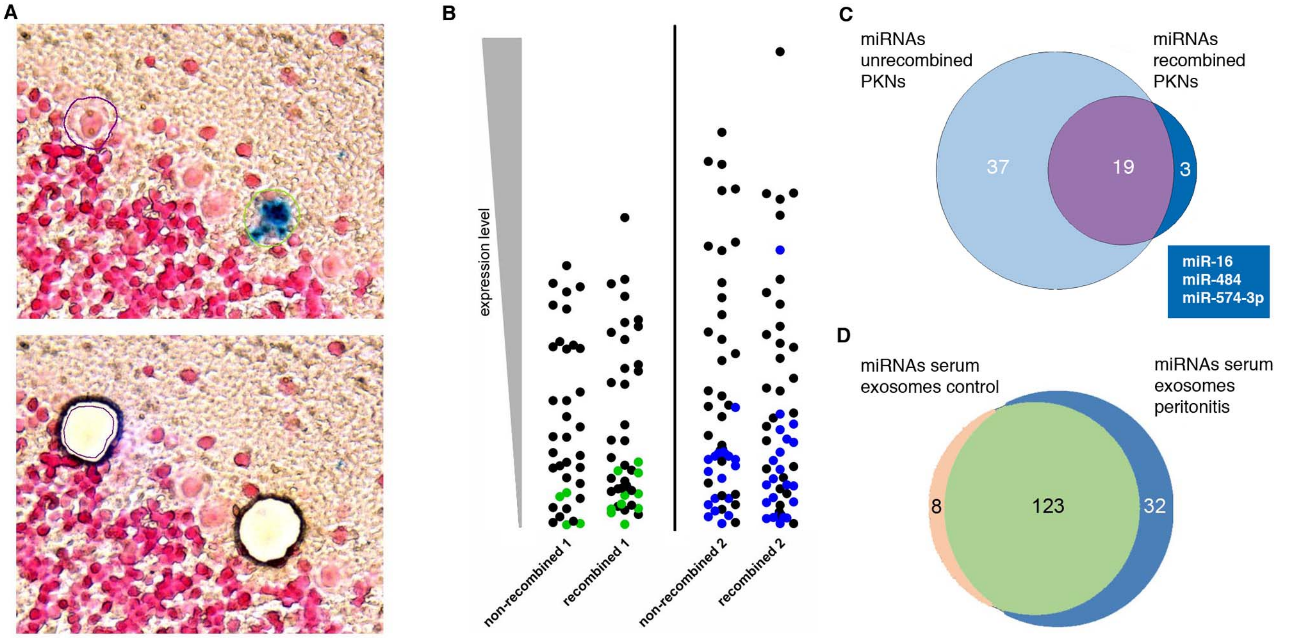

Figure 7. Recombined Purkinje neurons display a different miRNA profile compared to nonrecombined neighboring neurons. (A) Example for an X-Gal–positive neuron and corresponding nonrecombined Purkinje neuron that were collected by LCM for miRNA analysis. Cells were identified based on morphology and location in the Purkinje cell layer. Nonrecombined Purkinje neurons in the direct vicinity were cut out as references, depicted below. (B) Pairwise comparison of nonrecombined versus recombined Purkinje neurons from the same animal. Each symbol represents an individual miRNA; colored symbols represent miRNAs that could only be detected in one pool. Symbols representing higher expressed miRNAs are at the top; those representing lower expressed miRNAs are at the bottom of each column. The comparison of recombined versus nonrecombined PKNs from the same animals reveals significant differences in their miRNA profile. (C) Venn diagram depicting miRNAs specific for recombined PKNs. The three miRNAs displayed in the box are those that can be found in both samples of the recombined PKNs but not in any sample of the nonrecombined PKN. (D) EVs isolated from the serum of mice with peritonitis display a different miRNA content than those of healthy control animals.
doi:10.1371/journal.pbio.1001874.g007

Reporter Gene-Positive Neurons Are Present in Various Areas of the Brain

Heterotypic cell fusion in the brain induced by peripheral inflammatory injury has only been reported in Purkinje neurons [5,22,23]. However, because inflammation contributes to various neural pathologies such as epilepsy [24], neurodegenerative diseases [25,26], and sickness behavior [27], we tested whether other neuronal populations apart from Purkinje neurons could display Cre-mediated marker gene expression. Analyzing mice with an inflammation revealed different brain areas with recombined neurons. These were tyrosine hydroxylase (TH)-immunoreactive dopaminergic neurons in the substantia nigra/ventral tegmental area (SN/VTA) (Figure 6A,B). For this area, we could also observe recombined cells with a neuronal morphology that were negative for TH (Figure 6C), indicating loss of TH expression due to inflammation. Additionally we detected recombination in cortical neurons (Figure 6D–F) and in the granular cell layer of the hippocampus (Figure 6G–I). In the animals with an ECL, neurons in the hippocampal areas CA1 and CA3 were recombined as well as cells at the lesion site that were neither of a neuronal nor astrocytic lineage (Figure 6J–L). All neurons displaying marker gene expression contained only one nucleus, consistent with our observations in the cerebellum that cell fusion between hematopoietic cells and neurons is not the reason for the observed recombination events.

Next we wanted to test if an increase in recombination levels in Purkinje neurons is restricted to our injury model or constitutes a general response to any type of injury. Thus, we analyzed if a lesion that is specific for a selected group of neurons and that does not induce a systemic inflammation is capable of increasing recombination events in the Purkinje neuron population. To this end, we injected 1-methyl-4-phenyl-1,2,3,6-tetrahydropyridine (MPTP) into Vav-iCre-LacZ mice ($n = 3$). MPTP is converted to the toxin MPP+ (1-methyl-4-phenylpyridinium) in glial cells that is then released and taken up specifically via the dopamine transporter of dopaminergic neurons, leading to cell death by inhibiting mitochondrial complex I. Animals were intraperitoneally injected with MPTP (30 mg/Kg body weight per day) on 5 consecutive days. Two weeks after the last injection, animals were killed and their brains analyzed. MPTP treatment leads to a massive reduction in the number of dopaminergic neurons in the SN, more than in the VTA (Figure S3 A,B). As a result, we did not observe an increase in the number of recombined Purkinje neurons in the cerebellum comparable to levels seen after peripheral inflammation (53.3 ± 14.7 SD labeled Purkinje neurons per cerebellar hemisphere, $n = 3$). Therefore, an increase in recombined Purkinje neurons seems to be more injury-specific, particularly for a peripheral inflammation and not to limited mechanical damage, similar to findings in the context of cell fusion where mechanical trauma did not lead to an increase in heterokaryon formation [5]. Interestingly, we could detect numerous TH-negative LacZ-positive cells with neuronal morphology in the SN/VTA (Figure S3C–E). This could indicate an effect of a cell-type-specific lesion on a heightened signaling of blood-derived EVs, but we did not formally test this possibility.

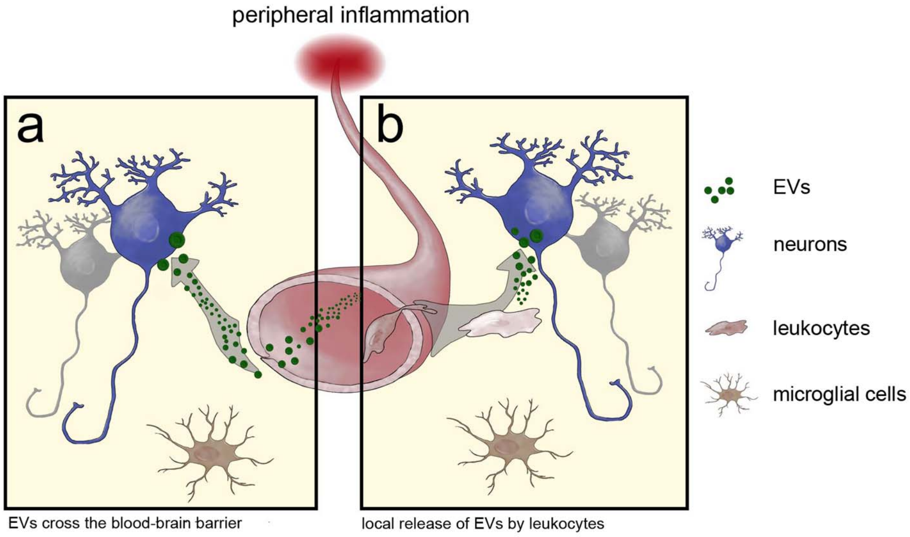

Figure 8. Model for EV transfer of RNA from immune cells to the brain. Possible modes of RNA transfer from blood to brain: (A) EVs are released from blood cells into the blood stream, where they can cross the BBB and fuse with neurons. (B) Alternatively, leukocytes enter the brain and only EVs released in short distance to the target cell are able to bind and release their content. This direct signaling of immune cells to the brain is independent of microglia.
doi:10.1371/journal.pbio.1001874.g008

Purkinje Neurons Display a Different miRNA Profile After Recombination

Finally, we wanted to test whether the EV transfer of Cre mRNA to Purkinje neurons is accompanied by other cellular changes that may indicate a more than transient physiological response. EVs and exosomes contain short noncoding RNAs including microRNAs (miRNAs), and it has been shown *in vitro* that functional miRNAs can be transferred by exosomes to target cells where they exert their effects on gene regulation [10,28]. Furthermore, miRNAs are abundant in the nervous system, and there is compelling evidence for a role of miRNAs in all aspects of neuronal function from development to plasticity in the adult brain [29]. Hence we asked whether we could observe differences in the miRNA profile in recombined Purkinje neurons compared to their nonrecombined counterparts. To this end, we induced peritonitis in two Vav-iCre-LacZ mice. Four days later, these animals were killed and their cerebella snap-frozen and serially cut. After brief fixation, sections were stained for X-Gal and screened for marker-positive Purkinje neurons on a microscope fitted for laser capture microdissection (LCM). Cells that were X-Gal–positive and clearly identifiable as Purkinje neurons based on morphology and location were cut out and collected. To obtain a reference population that was as similar as possible, for each recombined cell a corresponding neighboring X-Gal–negative Purkinje neuron (<5 neuron distance) was cut out and collected in a separate tube (Figure 7A). We collected 100 and 50 pairs of recombined/nonrecombined neurons. Material obtained in this way showed little RNA fragmentation as well as high specificity for Purkinje neurons (Table S2). Next, miRNAs were isolated from all four samples and analyzed by miRNA qPCR arrays. Pairwise comparison of the miRNAs from recombined neurons with their nonrecombined counterparts revealed significant differences in the presence or absence of various miRNAs (Figure 7B, Table S3) as well as changes in expression levels of common miRNAs (Figure S4). Comparing the miRNAs that were present in both sets of recombined neurons but not in any of the nonrecombined neurons yielded three miRNAs (Box in Figure 7C). Interestingly, of these miRNAs, miR-574-3p is highly expressed in myeloid cells and can be isolated from blood plasma [30] but is not present in previously published miRNA libraries specifically prepared from Purkinje neurons [31]. Additionally, we wanted to analyze whether an inflammation alters the composition of miRNAs contained in circulating serum exosomes. Therefore, we isolated serum exosomes from a group ($n = 20$) of mice 24 h after induction of peritonitis as well as from an untreated control group ($n = 19$). The vesicle preparations for each group were separated by sucrose density gradient ultracentrifugation as described above, and only the exosomal fractions were used for further analysis. miRNAs were isolated from both preparations and analyzed on a miRNA qPCR array. The miRNA profiles of the two samples indicated significant changes in miRNA composition in response to an inflammatory stimulus (Figure 7D, Table S4). All of the three miRNAs identified specifically in the recombined Purkinje neurons above were contained in the exosomal miRNA population.

Discussion

Our current study reveals a previously unrecognized mechanism behind how the hematopoietic system may influence cellular processes in the brain in response to inflammation. Our results show that EVs, including exosomes, are released from hematopoietic cells and transfer functional RNA to cells of multiple tissues, including Purkinje neurons in the brain. This EV transfer is a relatively rare event under nonpathological conditions but can rapidly increase by orders of magnitude after peripheral inflammation. It is not restricted to Purkinje neurons in the cerebellum but includes multiple other neuronal types, notably dopaminergic neurons in the SN. Importantly, Purkinje neurons that receive EV RNA, measured by Cre-mediated induction of reporter gene expression, display a different miRNA profile compared to their nonrecombined counterparts, indicating physiologically relevant changes.

Exosomes are increasingly recognized as an agent that can transmit genetic information between cells, but their analysis has

largely been restricted to *in vitro* studies. Therefore, the finding that functional Cre mRNA can be transferred by exosomes leading to loxP excision in target cells will serve as a valuable tool to understanding the physiological role of exosomes *in vivo*. However, our results also caution against a lateral transfer of Cre mRNA as a possible confounding factor when using the Cre-lox system for lineage analysis *in vivo*.

In this study, we used two transgenic mouse lines expressing Cre recombinase in hematopoietic cells and one additionally in the endothelial lineage. Although in the Vav-iCre mouse Cre is constitutively expressed in virtually all hematopoietic cells [6,32], Tie-2 expression in the adult animal is generally associated with endothelial cells and tumor-infiltrating proangiogenic monocytes [33]. However, its expression has also been described or suggested in other hematopoietic cell types [34]. Additionally, cells may up-regulate Tie-2 in response to inflammatory injury in yet other subpopulations. Based on the similarity of marker gene expression in both mouse models, we rule out endothelial cells as a source for EVs. In contrast, mouse lines expressing Cre under the nonhematopoietic promoters for nestin, myelin oligodendrocyte glycoprotein, and glycine transporter 2 did not show any marker-gene–positive Purkinje neurons, further arguing against unspecific Cre expression (unpublished data). Importantly, the fact that we do not observe microglia expressing the marker gene in both animal models demonstrates that a vesicular transfer to neurons does not involve the participation of this cell type. Neither are microglia the target of peripheral blood-derived secreted membrane vesicles, although our results from the intracerebellar injection of Cre RNA-containing exosomes as well as published work [35] suggest that microglia can in principle take up EVs.

Irrespective of the cell of origin, horizontal transfer of genetic material by EVs may occur in two fundamentally different, but not necessarily mutually exclusive, ways: EV release by hematopoietic cells into the blood stream increases in response to a peripheral inflammation. They then reach the different organs via the blood supply where they are taken up by local cells (Figure 8A). The specificity of this uptake is unknown, but work by others generally shows that EV uptake can be membrane-receptor-mediated [36] or occur by phagocytosis [37]. The essential question in this model though would be how EVs are able to pass through the BBB. As a second possibility, leukocytes enter the brain and secrete exosomes that are taken up by cells in close apposition to the secreting cells (Figure 8B). The fact that we do not find any marker-gene–positive leukocytes in the cerebellar parenchyma together with the presence of Cre mRNA-containing vesicles in the peripheral blood of Vav-iCre mice suggests to us that the first route, via EVs in the circulation, is the more likely scenario. Together with the results from direct injection of Cre-containing EVs into the cerebellum, this also argues against other conceivable mechanisms enabling a local Cre transfer—for example, via tunneling nanotubes or gap junctions. However, our observations do not rule out instances of such a local transfer by leukocytes after entering the brain. Particularly for more severe injuries and pathologies such as lethal irradiation and induced autoimmune encephalitis that were used in studies of cell fusion, a leukocyte entry into the CNS parenchyma is known [38]. Our results from the ECL indeed indicate that local transfer of EV Cre mRNA also could occur. The similarities in recombination pattern, frequency, and induction by inflammation compared to the phenomenon of heterotypic cell fusion are intriguing. We reason that in both cases lipid bilayer membranes need to fuse and that the fusion of whole cells might represent an extreme case of vesicle-cell fusion, triggered by the experimental conditions. The fact that none of the recombined neurons in our inflammation models was binucleated underlines the fact that an inflammatory stimulus as such is not sufficient to lead to cellular fusion, in contrast to reports using various experimental approaches to generate blood chimeras [5,14].

The transfer of functional genetic material via EVs and concomitant changes in miRNA content of the receiving cells that we observe suggests a novel way in how the hematopoietic compartment may interact with multiple organs, including the brain, which deserves further study. Peripheral infections and inflammation have been linked to processes such as cancer [39], neurodegenerative diseases [1], and sickness behavior—that is, changes in cognitive functions due to peripheral infections [27]. So far, this link has been mainly explained as cytokine-mediated effects. Increasing evidence points to a central role of miRNAs in regulating neuronal function. In this respect our data open the possibility of a simultaneous lateral transfer of multiple miRNA species from blood cells via EVs as an additional factor contributing to inflammation-induced reactions. Interestingly, although the miRNA profile of nonrecombined Purkinje neurons from our analysis almost completely overlaps with published libraries specifically prepared from Purkinje neurons based on pcp2 expression [31], we find significant differences in comparison to the miRNAs contained in the recombined Purkinje neurons (Table S5). EV-derived miRNAs could act as a general stabilizer of transcription to compensate effects of cellular stress [40]. A recent report of oligodendrocyte-derived EVs increasing resistance of neurons against different types of stress *in vitro* could be a reflection of this function [41]. With regard to specific functions of individual miRNAs, interestingly miRNA-574-3p that we detect only in recombined Purkinje neurons as well as in exosomes from peripheral blood regulates and in turn is regulated by TAR DNA-binding protein 43 (TDP-43) [42,43]. TDP-43 is a heterogeneous nuclear ribonucleoprotein involved in RNA processing and stability and has a central role in neurological diseases such as amyotrophic lateral sclerosis and frontotemporal lobar degeneration as the main component of brain inclusions [44]. In the context of these diseases, miR-574-3p could be detected in the serum but also in cerebrospinal fluid, with a down-regulation in cerebrospinal fluid of affected patients [43]. miR-484 has been reported to regulate mitochondrial fission [45], and mitochondrial fission is involved in oxidative stress, apoptosis, and many neurological diseases. Finally miR-16 has been demonstrated to be a repressor for the serotonin transporter and is implicated in the therapeutic action of the antidepressant fluoxetine [46]. Furthermore it is associated with neuronal differentiation [47]. Therefore, published evidence about the miRNAs that we identify in recombined Purkinje neurons is consistent with a functional role in neurons. Although we want to stress that our results are not formal proof for a direct transfer of miRNAs via EVs and that the presence of these miRNAs could also be the result of an indirect regulation, they nonetheless highlight the possibility of how miRNAs in body fluids may reach target cells in various organs, specifically neurons in the brain.

Recently, it has been demonstrated that peripherally injecting exosomes containing exogenous siRNA against GAPDH and engineered to specifically target neurons could indeed lead to down-regulation of their target gene in the brain [28]. Our data suggest that a more widespread gene regulation via changes in miRNA content in neurons naturally occurs *in vivo*.

Finally, there is increasing evidence that key proteins in the pathology of neurodegenerative diseases such as α-synuclein or tau can associate with exosomes [48,49] or can be intercellularly transferred by as yet unknown mechanisms in Parkinson's disease [50,51]. In this context, our findings extend the possibility of a

spreading of diseases between tissues with the involvement of EVs and provide an accessible method for further studies in this exiting field.

Materials and Methods

Animals

All animal work has been conducted according to relevant institutional guidelines of the Frankfurt University Medical School (ethical permission Gen. Nr. F94/19), and the Philipps University Marburg. Vav-iCre, and Tie2-Cre transgenic mice used in this study have been previously published [6–8]. Both Cre lines were crossbred with Rosa26-LacZ (JAX-mice stock number 003309) or Rosa26-EGFP Cre reporter mice (JAX-mice stock number 004077). Genotyping was performed as described previously. In brief, PCR of tail biopsy lysates was performed using the following primers: iCre F, 5′-CTCTGACAGATGCCAGGACA-3′, R, 5′-ACACCATTCTTTCTGACCCG-3′; Cre F, 5′-GCCTGCATTACCGGTCGATGCAACGA-3′, R, 5′-GTGGCAGATGGCGCGGCAACACCATT-3′; and LacZ 1 F, 5′-GCGAAGAGTTTGTCCTCAACC-3′, 2 F, 5′-GGAGCGGGAGAAATGGATATG-3′, R, 5′-AAAGTCGCTCTGAGTTGTTAT-3′. Amplificates were visualized on a 2% agarose gel.

Inflammation Models

To induce peripheral tumors, 250,000 LLC2s resuspended in 200 µl of PBS were injected subcutaneously into both flanks. Tumors were let to grow for a maximum of 12 d. Peritonitis was induced by a single i.p. injection of thioglycolate broth (Merck) (1 ml 3% in PBS). Animals were killed 4 d after injection.

ECL

Unilateral transection of the left perforant path was performed as described previously [52]. Briefly the left medial entorhinal cortex was lesioned using a 2 mm broad stainless steel blade. The medial edge of the knife was adjusted to the following coordinates as measured from the λ: anterior–posterior, 0.4 mm; lateral, 1.2 mm; dorsoventral, down to the base of the skull. Animals were killed 4 d after the surgery.

Slice Culture Experiments

Brains from Vav-iCre/Tie2-Cre-ROSA26Lacz/GFP mice were removed immediately after cervical dislocation and placed in ice-cold culture medium (DMEM, 10% FCS with added antibiotics). Slice cultures were prepared as described [53]. Briefly brain hemispheres were embedded in 2% low melting point agarose and cut in 300 µm sections on a vibratome (Leica). The other hemisphere was directly placed in 4% paraformaldehyde (PFA) and stored for later comparison of recombined cells. Two to three slices were transferred to one membrane insert (PICM0RG50; Millipore) in six-well plates and incubated in a humidified 5% CO_2 atmosphere at 37°C. On the next day, culture medium was replaced with serum-free Neurobasal medium, 2% B27, HEPES buffer, and antibiotics. For an injury, slices were cut transversally with a scalpel blade. Three days later, slices were fixed on their membrane inserts with 4% PFA at 4°C overnight.

Tissue Processing

Deeply anesthetized mice were perfused transcardially with PBS followed by 4% cold PFA in PBS. All organs were postfixed in 4% PFA in PBS for 24 h. For cryosectioning, organs were cryoprotected in 15% sucrose for an additional 24 h before they were embedded and sectioned (10–12 µm) as a frozen block. Sagittal cerebellar sections (50–60 µm) were cut on a vibratome (Leica) and kept in PBS at 4°C.

Hematopoietic Cell Cultures

Whole blood withdrawal was performed on deeply anesthetized Vav-iCre mice via cardiac puncture. After blood withdrawal, mice were killed by cervical dislocation. Legs were removed in order to obtain BMCs. Erythrocytes were depleted using red blood cell lysis buffer (Roche). Cells were flushed through a 70 µm cell strainer and washed with PBS before culturing them in serum-free IMDM supplemented with 5% Panexin-NT (PanBiotech), 100 µg streptomycin, 100 U/ml penicillin, glutamine, and 200 ng/ml LPS. Cells were seeded at a density of 5×10^6 cells/ml for 48–96 h at 37°C and 5% CO_2.

EV Preparations

From hematopoietic cell culture, the supernatant was collected and processed by differential centrifugation as described [54], with additional filtering (0.22 µm) of the supernatant preceding the ultracentrifugation steps. For sucrose density gradient fractionation, vesicles were loaded on top of a stepwise sucrose gradient at the following concentrations: 21M, 1.3 M, 1.16 M, 0.8 M, 0.5 M, and 0.25 M in TBS as described previously [54]. The gradient was centrifuged for 2.5 h at $100{,}000\times g$ using a Beckman SW40 Rotor. Twelve 1 ml fractions were collected from the top of the gradient, diluted with PBS, and subjected to ultracentrifugation. The pellet was taken up and used for RT-PCR.

For blood plasma and serum, peripheral blood was drawn by heart puncture from deeply anesthetized mice. Vesicle preparation was performed similar to preparations from the cell culture supernatant with the necessary elongation steps for more viscous liquids as described [11]. For blood serum, vesicle preparations were separated by a sucrose density gradient as described above, and only the exosomal fractions were used for further analysis.

Human Specimens

Formalin-fixed and paraffin-embedded tissue of human cerebella from the Edinger Institute archive was cut into 4 µm serial sections and stained after rehydration by serial incubation in xylol (2×10 min) and ethanol (98% to water, 5 min each). Every fifth section was analyzed to avoid counting the same neuron twice.

Staining and Microscopic Analysis

Antibodies that were used were anti-mouse Calbindin (mouse monoclonal, Sigma), anti–β-Gal (mouse monoclonal, Promega; rabbit polyclonal, MP Biomedicals), Alexa 488–conjugated anti-NeuN (mouse monoclonal, Chemicon), anti-TH (rabbit polyclonal or mouse monoclonal, both Chemicon), anti-Iba1 (rabbit polyclonal, Wako), Alexa 488–conjugated anti-GFP (rabbit polyclonal, Invitrogen), polyclonal rabbit anti–glial fibrillary acidic protein (GFAP) (Dako), anti-human CD68, CD15, and leukocyte common antigen (LCA) (all mouse monoclonal, Dako). For light microscopic staining, polylink and peroxidase label (DCS) were used in combination with the AEC substrate pack (BioGenex) followed by a hemalum counterstaining. Light microscopic images were recorded on an Olympus 80i microscope; fluorescent images were taken on a confocal LSM Nikon TE2000-E microscope. Images were processed using the EC-C1 3.60 software and ImageJ (NIH). Figures were mounted in Adobe Photoshop CS4.

Purkinje Neuron Quantitation

For the quantitation of cerebellar Purkinje neurons, all sections from one cerebellar hemisphere were stained for LacZ or GFP,

and recombined Purkinje neurons were counted in every other section equaling one quarter of the population.

RNA and Protein Analysis

RNA was purified using Qiagen RNeasy Micro Kit, according to the manufacturer's instructions. cDNA synthesis was performed with SuperScipt III reverse transcriptase (RT) (Invitrogen) and OligodT18 Primers (Fermentas). Residual DNA contamination was excluded by controls in which RT was omitted during cDNA synthesis. For iCre mRNA detection, a heminested PCR was performed with 5 µl of vesicle cDNA preparation and Primers FORWARD-out and REVERSE (FORWARD-out, 5′-GTGGGAGAATGCTGATCCACA-3′; REVERSE, 5′-ACAC-CATTCTTTCTGACCCG-3′) in the first reaction followed by the second PCR using 2 µl of the amplificate and FORWARD-in (5′-GGTTACCAAGCTGGTGGAGA -3′) and REVERSE. PCR products were visualized on a 3% agarose gel. For protein analysis, acetone was added to the vesicle containing sucrose fractions. Precipitates were boiled in SDS sample buffer to further perform SDS/PAGE and Western blot analysis with antibodies to anti-mouse CD9 (R&D Systems, clone 139712), anti-mouse ADAM10 (sc-18869, Santa Cruz), and anti–Cre-recombinase (Covance, NMS-106P).

Electron Microscopy

Vesicle preparations were fixed and stained as described [11] with minor alterations. The uranyl-oxalate (step 6) was substituted with 2% of uranyl-acetate, and the ratio of methylcellulose to uranyl-acetate was changed to 1% each (step 7). Grids were analyzed using Tecnai Spirit BioTWIN electron microscope (FEI, Netherlands) at 120 kV. Pictures were taken with an eagle bottom-mount CCD camera.

In Vivo Experiments

Secreted membrane vesicle preparations obtained from hematopoietic cell culture were resuspended in HEPES buffer and injected i.v. (200 µl) or directly into cerebellar hemispheres of anesthetized Cre-reporter mice (1 µl). Injections of 1 µl Cre recombinase protein (New England Biolabs) (1 U/µl or 91 n g/µl) or lysate from Cre-expressing BMCs served as controls. One or two intracerebellar injections were performed per hemisphere with 1 µl each at 6.2 mm caudal and 2 mm lateral to bregma and 1.5 mm below the dura mater. Mice were kept for 4–5 d until they were sacrificed for subsequent analysis as above. All sections from the injected cerebellar hemisphere were stained with an anti–β-Gal antibody with AEC Kit, and recombined Purkinje neurons were counted in every section.

Impedance Measurements

Impedance measurements were performed with a cellZscope device (nanoAnalytics) as described previously [21] using the endothelial cell line bEnd5. After reaching confluence, indicated by a plateau in the TEER and Ccl values, EVs prepared from the supernatant of 10×10^7 BMCs after 3 d in culture were added to the transwell units. The cell culture supernatant after the ultracentrifugation step was used at the same volume as the control (100 µl of EV preparation/supernatant diluted in 900 µl endothelial cell medium). Measurements were performed three times in quadruplicates with EVs from three separate preparations.

MPTP Experiments

Mice were intraperitoneally injected 5 times (24 h apart) with MPTP (30 mg/kg; Sigma; 3 mg/ml saline) or saline (10 ml/kg) as the control. Animals were sacrificed and brains were carefully removed 15 d after the last injection.

Cell Transplantation and Irradiation

BMCs of donor mice were extracted by flushing femurs and tibias with chilled PBS supplemented with 1% bovine serum albumin (PBS/BSA) into 35 mm tissue culture dishes on ice. BMCs were washed in cold PBS/BSA, suspended in PBS/BSA to a final concentration of 25×10^6/ml, and kept on ice until further use. Recipient mice were lethally gamma-irradiated with a single dose of 9.5 Gy using a Cesium source with a dose rate of 1 Gy/min. Within 3 h after irradiation, 5×10^6 BMCs were injected into the lateral tail vein. After transplantation, mice were kept in filtertop boxes and received antibiotic prophylaxis (0.025% Baytril, Bayer) p.o. in drinking water until engraftment. Mice were analyzed 5 wk or 2 mo after transplantation, respectively.

For adoptive transfer, spleen and lymph nodes of donor mice with peritonitis induced the previous day were dissociated into PBS, and 20×10^6 cells were injected into the lateral tail vein. Animals were analyzed after 10 d.

Laser Microdissection of Single Purkinje Neurons

Brains from Vav-iCre-LacZ mice were rapidly removed from the cranium 4 d after induction of peritonitis, flash-frozen in tissue freezing medium on dry ice, and stored at −80°C until sectioning. For X-Gal staining, cerebellar parasagittal cryostat sections (8 µm) were collected on polyethylene-naphthalene RNase-free slides (Leica Microsystems). The sections were fixed in 4% PFA in PBS for 20 min and briefly washed in RNase-free water. Tissue sections were then fixed in 70% ethanol for 5 min, washed in RNase-free water for 5 min, and stained in X-Gal reaction buffer for approximately 16–20 h at 37°C. For cerebellar histology, brain slices were counterstained with nuclear fast red staining solution (Sigma). A Leica DM6500 LMD system (Leica Microsystems) was used to dissect single X-Gal–positive Purkinje cells separately from adjacent X-Gal–negative Purkinje cells as controls. Cell pools of 50 and 100 pairs of recombined/nonrecombined Purkinje cells per animal were collected for further analysis as well as an approximate area (size, ~100,000 μm^2) from the adjacent granule cell layer (GCL) as control. Total RNA was isolated from the microdissected PC and GCL using RNeasy Plus Micro Kit (Qiagen). RNA integrity analysis using the Agilent 2100 bioanalyzer and RNA 6000 Pico LabChip Kit (Agilent Technologies) revealed highly intact RNA (RIN, 8.2–8.8). Total RNA was reverse transcribed using the High Capacity cDNA Reverse Transcription Kit (Applied Biosystems). Real-time reverse transcription polymerase chain reaction (RT-qPCR) of distinct target genes (Table S2) was performed on a StepOnePlus Real-Time PCR System (Applied Biosystems) using EXPRESS SYBRGreenER qPCR SuperMix (Invitrogen) or TaqMan Fast Universal PCR Master Mix (Applied Biosystems) with TaqMan Gene Expression Assays (Applied Biosystems).

Screening of miRNA by Quantitative Real-Time PCR

miRNA from LCM Purkinje neurons or from serum exosomes was isolated using the miRNeasy Micro Kit (Qiagen) according to the manufacturer's protocol. The extracted miRNA was diluted in 15 µl H_2O. Quantitative miRNA expression analysis was performed using real-time PCR (LightCycler 480 Real-Time PCR System, Roche Applied Science). To screen for deregulated miRNA in the LCM Purkinje neurons or serum exosomes, the miRCURY LNA Universal microRNA Ready-to-Use PCR panel (Exiqon) was used according to the manufacturer's instructions. Prior to the analysis, plate-specific effects were normalized using

the reference RNA included. Cp values from recombined Purkinje neurons were then compared to the Cp values measured in nonrecombined Purkinje neurons.

Supporting Information

Figure S1 Analysis of exosomes derived from Vav1-Cre mice. (A) In this analysis, Cre mRNA is only present in the pooled exosomal fractions 2–7. The nonexosomal vesicle fractions (8–12) contain no detectable amounts of Cre mRNA. (B) Nanosight analysis of vesicle size distribution in exosomes isolated from blood plasma (in red) or from the supernatant of cultivated blood cells (in blue) from Vav1-iCre mice. An increase in larger size vesicles can be observed for the cell culture supernatant-derived EVs, indicating an increase in membrane blebs/apoptotic bodies or clustering of vesicles in culture.
(TIF)

Figure S2 No evidence for elevated numbers of binucleated Purkinje neurons in cerebella of human patients with inflammation. (A) Rare occurrence of a second nucleus-like inclusion in a Purkinje neuron of a human cerebellum from a patient with a diagnosed inflammation ($n<4$ for all neurons in all analyzed cases). In these rare cases, the possible nuclear structures were all of very small size (4 μm) and clearly distinct from the large neuronal nuclei typical for Purkinje neurons. Purkinje neurons containing two similar neuronal nuclei indicating heterotypic cell fusion and subsequent reprogramming of the hematopoietic nucleus were never observed. Archived brain tissue of each patient was tested for inflammatory lesions with the combination of markers CD15 for granulocytes (B), CD68 for activated microglia (C), and LCA for mononuclear leucocytes (D).
(TIF)

Figure S3 Frequent occurrence of recombined TH-negative cells with neuronal morphology in the SN/VTA in the MPTP mouse model of Parkinson's disease. Reduction in the number of TH-positive cells in the SN after MPTP treatment (B) as compared to NaCl (A). (C and D) TH-negative cell with neuronal morphology next to TH-positive dopaminergic neurons in the SN. (E) Several TH-negative cells with neuronal morphology expressing the reporter gene in the midbrain in brains of MPTP-treated mice.
(TIF)

Figure S4 Heatmap of miRNA expression in recombined–nonrecombined pairs of Purkinje neurons. Linear fold changes between recombined and nonrecombined neurons are listed. Values >1.2 and <0.83 are colored red and blue, respectively.
(TIF)

Table S1 Human brain sections derived from patients with inflammatory injuries analyzed for binucleated Purkinje neurons. Cerebella from adult human brains, all from patients clinically diagnosed with inflammations, were analyzed for Purkinje neurons containing two nuclei. According to published data and our own results obtained from mouse brains, the inflammatory lesion can increase the percentage of fused or recombined Purkinje neurons from 0.2% to 14% of the total population. Assuming similar quantities in the human brain, we should have observed between 700 and 50,000 binucleated Purkinje neurons in all cases combined.
(DOCX)

Table S2 miRNAs detectable in recombined and nonrecombined Purkinje neurons. miRNAs identified by qPCR array analysis with a Cp<36 from material isolated from microdissected Purkinje neurons based on the presence or absence of X-Gal staining.
(DOCX)

Table S3 List of miRNAs associated with serum exosomes. List of all miRNAs identified by qPCR array analysis with a Cp<36 contained in the exosomal fraction (2–7) of blood sera from mice with or without an inflammation.
(PDF)

Table S4 Assessment of RNA quality obtained by LCM. Estimating RNA fragmentation and specificity by testing for the expression of genes that are specific for Purkinje or other types of neurons as well as glial cells. Expression levels of Cp>35 were considered to be not expressed.
(PDF)

Table S5 Comparison of microdissected Purkinje neuron miRNAs with published miRNA profiles. Comparison of miRNAs detected in microdissected Purkinje neurons with miRNAs in three libraries (PKN 1–3, >10 pm reads) obtained from healthy animals in He et al. [31]. The total number of identified miRNAs contained in each library is given in brackets.
(DOCX)

Acknowledgments

We thank B. Berninger, I. Dikic, J. Frisén, J. Priller, and M. Schäfer for comments on the manuscript and discussion; M. Mittelbronn for help with archived human brain material; S. Prokosch for excellent technical assistance; S. Gelhardt for animal breeding; and S. Mükusch for schematic drawings.

Author Contributions

The author(s) have made the following declarations about their contributions: Conceived and designed the experiments: SK DD KD CD BA GH HS PA SM. Performed the experiments: KR SK MD AR JSc JSt JM DK KD CD BL SM. Analyzed the data: KR SK MD AR JSc DD JSt JM KD GH KP HS PA SM. Contributed reagents/materials/analysis tools: KP. Wrote the paper: PA SM.

References

1. Glass CK, Saijo K, Winner B, Marchetto MC, Gage FH (2010) Mechanisms underlying inflammation in neurodegeneration. Cell 140: 918–934.
2. Terada N, Hamazaki T, Oka M, Hoki M, Mastalerz DM, et al. (2002) Bone marrow cells adopt the phenotype of other cells by spontaneous cell fusion. Nature 416: 542–545.
3. Ying QL, Nichols J, Evans EP, Smith AG (2002) Changing potency by spontaneous fusion. Nature 416: 545–548.
4. Alvarez-Dolado M, Pardal R, Garcia-Verdugo JM, Fike JR, Lee HO, et al. (2003) Fusion of bone-marrow-derived cells with Purkinje neurons, cardiomyocytes and hepatocytes. Nature 425: 968–973.
5. Johansson CB, Youssef S, Koleckar K, Holbrook C, Doyonnas R, et al. (2008) Extensive fusion of haematopoietic cells with Purkinje neurons in response to chronic inflammation. Nat Cell Biol 10: 575–583.
6. de Boer J, Williams A, Skavdis G, Harker N, Coles M, et al. (2003) Transgenic mice with hematopoietic and lymphoid specific expression of Cre. Eur J Immunol 33: 314–325.
7. Nern C, Wolff I, Macas J, von Randow J, Scharenberg C, et al. (2009) Fusion of hematopoietic cells with Purkinje neurons does not lead to stable heterokaryon formation under noninvasive conditions. J Neurosci 29: 3799–3807.
8. Koni PA, Joshi SK, Temann UA, Olson D, Burkly L, et al. (2001) Conditional vascular cell adhesion molecule 1 deletion in mice: impaired lymphocyte migration to bone marrow. J Exp Med 193: 741–754.
9. Thery C, Ostrowski M, Segura E (2009) Membrane vesicles as conveyors of immune responses. Nat Rev Immunol 9: 581–593.

10. Valadi H, Ekstrom K, Bossios A, Sjostrand M, Lee JJ, et al. (2007) Exosome-mediated transfer of mRNAs and microRNAs is a novel mechanism of genetic exchange between cells. Nat Cell Biol 9: 654–659.
11. Thery C, Amigorena S, Raposo G, Clayton A (2006) Isolation and characterization of exosomes from cell culture supernatants and biological fluids. Curr Protoc Cell Biol Chapter 3: Unit 3 22.
12. Fevrier B, Raposo G (2004) Exosomes: endosomal-derived vesicles shipping extracellular messages. Curr Opin Cell Biol 16: 415–421.
13. Keller S, Konig AK, Marme F, Runz S, Wolterink S, et al. (2009) Systemic presence and tumor-growth promoting effect of ovarian carcinoma released exosomes. Cancer Lett 278: 73–81.
14. Nygren JM, Liuba K, Breitbach M, Stott S, Thoren L, et al. (2008) Myeloid and lymphoid contribution to non-haematopoietic lineages through irradiation-induced heterotypic cell fusion. Nat Cell Biol 10: 584–592.
15. Singec I, Snyder EY (2008) Inflammation as a matchmaker: revisiting cell fusion. Nat Cell Biol 10: 503–505.
16. Distler JH, Jungel A, Huber LC, Seemayer CA, Reich CF, 3rd, et al. (2005) The induction of matrix metalloproteinase and cytokine expression in synovial fibroblasts stimulated with immune cell microparticles. Proc Natl Acad Sci U S A 102: 2892–2897.
17. Bala S, Petrasek J, Mundkur S, Catalano D, Levin I, et al. (2012) Circulating microRNAs in exosomes indicate hepatocyte injury and inflammation in alcoholic, drug-induced, and inflammatory liver diseases. Hepatology 56: 1946–1957.
18. Prang P, Del Turco D, Deller T (2003) Associational sprouting in the mouse fascia dentata after entorhinal lesion in vitro. Brain Res 978: 205–212.
19. Potolicchio I, Carven GJ, Xu X, Stipp C, Riese RJ, et al. (2005) Proteomic analysis of microglia-derived exosomes: metabolic role of the aminopeptidase CD13 in neuropeptide catabolism. J Immunol 175: 2237–2243.
20. Ginhoux F, Greter M, Leboeuf M, Nandi S, See P, et al. (2010) Fate mapping analysis reveals that adult microglia derive from primitive macrophages. Science 330: 841–845.
21. Paolinelli R, Corada M, Ferrarini L, Devraj K, Artus C, et al. (2013) Wnt activation of immortalized brain endothelial cells as a tool for generating a standardized model of the blood brain barrier in vitro. PLoS ONE 8: e70233.
22. Priller J, Persons DA, Klett FF, Kempermann G, Kreutzberg GW, et al. (2001) Neogenesis of cerebellar Purkinje neurons from gene-marked bone marrow cells in vivo. J Cell Biol 155: 733–738.
23. Weimann JM, Johansson CB, Trejo A, Blau HM (2003) Stable reprogrammed heterokaryons form spontaneously in Purkinje neurons after bone marrow transplant. Nat Cell Biol 5: 959–966.
24. Vezzani A, French J, Bartfai T, Baram TZ (2011) The role of inflammation in epilepsy. Nat Rev Neurol 7: 31–40.
25. Sastre M, Richardson JC, Gentleman SM, Brooks DJ (2011) Inflammatory risk factors and pathologies associated with Alzheimer's disease. Curr Alzheimer Res 8: 132–141.
26. Whitton PS (2007) Inflammation as a causative factor in the aetiology of Parkinson's disease. Br J Pharmacol 150: 963–976.
27. Dantzer R, O'Connor JC, Freund GG, Johnson RW, Kelley KW (2008) From inflammation to sickness and depression: when the immune system subjugates the brain. Nat Rev Neurosci 9: 46–56.
28. Alvarez-Erviti L, Seow Y, Yin H, Betts C, Lakhal S, et al. (2011) Delivery of siRNA to the mouse brain by systemic injection of targeted exosomes. Nat Biotechnol 29: 341–345.
29. Im HI, Kenny PJ (2011) MicroRNAs in neuronal function and dysfunction. Trends Neurosci 35: 325–334.
30. Pritchard CC, Kroh E, Wood B, Arroyo JD, Dougherty KJ, et al. (2012) Blood cell origin of circulating microRNAs: a cautionary note for cancer biomarker studies. Cancer Prev Res (Phila) 5: 492–497.
31. He M, Liu Y, Wang X, Zhang MQ, Hannon GJ, et al. (2012) Cell-type-based analysis of microRNA profiles in the mouse brain. Neuron 73: 35–48.
32. Ogilvy S, Elefanty AG, Visvader J, Bath ML, Harris AW, et al. (1998) Transcriptional regulation of vav, a gene expressed throughout the hematopoietic compartment. Blood 91: 419–430.
33. De Palma M, Venneri MA, Galli R, Sergi Sergi L, Politi LS, et al. (2005) Tie2 identifies a hematopoietic lineage of proangiogenic monocytes required for tumor vessel formation and a mesenchymal population of pericyte progenitors. Cancer Cell 8: 211–226.
34. Puri MC, Bernstein A (2003) Requirement for the TIE family of receptor tyrosine kinases in adult but not fetal hematopoiesis. Proc Natl Acad Sci U S A 100: 12753–12758.
35. Fitzner D, Schnaars M, van Rossum D, Krishnamoorthy G, Dibaj P, et al. (2011) Selective transfer of exosomes from oligodendrocytes to microglia by macropinocytosis. J Cell Sci 124: 447–458.
36. El-Andaloussi S, Lee Y, Lakhal-Littleton S, Li J, Seow Y, et al. (2012) Exosome-mediated delivery of siRNA in vitro and in vivo. Nat Protoc 7: 2112–2126.
37. Feng D, Zhao WL, Ye YY, Bai XC, Liu RQ, et al. (2010) Cellular internalization of exosomes occurs through phagocytosis. Traffic 11: 675–687.
38. Hickey WF, Hsu BL, Kimura H (1991) T-lymphocyte entry into the central nervous system. J Neurosci Res 28: 254–260.
39. Grivennikov SI, Greten FR, Karin M (2010) Immunity, inflammation, and cancer. Cell 140: 883–899.
40. Schratt G (2009) Fine-tuning neural gene expression with microRNAs. Curr Opin Neurobiol 19: 213–219.
41. Fruhbeis C, Frohlich D, Kuo WP, Amphornrat J, Thilemann S, et al. (2013) Neurotransmitter-triggered transfer of exosomes mediates oligodendrocyte-neuron communication. PLoS Biol 11: e1001604.
42. Kawahara Y, Mieda-Sato A (2012) TDP-43 promotes microRNA biogenesis as a component of the Drosha and Dicer complexes. Proc Natl Acad Sci U S A 109: 3347–3352.
43. Freischmidt A, Muller K, Ludolph AC, Weishaupt JH (2013) Systemic dysregulation of TDP-43 binding microRNAs in amyotrophic lateral sclerosis. Acta Neuropathol Commun 1: 42.
44. Baralle M, Buratti E, Baralle FE (2013) The role of TDP-43 in the pathogenesis of ALS and FTLD. Biochem Soc Trans 41: 1536–1540.
45. Wang K, Long B, Jiao JQ, Wang JX, Liu JP, et al. (2012) miR-484 regulates mitochondrial network through targeting Fis1. Nat Commun 3: 781.
46. Baudry A, Mouillet-Richard S, Schneider B, Launay JM, Kellermann O (2010) miR-16 targets the serotonin transporter: a new facet for adaptive responses to antidepressants. Science 329: 1537–1541.
47. Aranha MM, Santos DM, Xavier JM, Low WC, Steer CJ, et al. (2010) Apoptosis-associated microRNAs are modulated in mouse, rat and human neural differentiation. BMC Genomics 11: 514.
48. Emmanouilidou E, Melachroinou K, Roumeliotis T, Garbis SD, Ntzouni M, et al. (2010) Cell-produced alpha-synuclein is secreted in a calcium-dependent manner by exosomes and impacts neuronal survival. J Neurosci 30: 6838–6851.
49. Saman S, Kim W, Raya M, Visnick Y, Miro S, et al. (2012) Exosome-associated tau is secreted in tauopathy models and is selectively phosphorylated in cerebrospinal fluid (CSF) in early Alzheimer's disease. J Biol Chem 287(6): 3842–3849.
50. Clavaguera F, Bolmont T, Crowther RA, Abramowski D, Frank S, et al. (2009) Transmission and spreading of tauopathy in transgenic mouse brain. Nat Cell Biol 11: 909–913.
51. Dunning CJ, Reyes JF, Steiner JA, Brundin P (2011) Can Parkinson's disease pathology be propagated from one neuron to another? Prog Neurobiol 97(2): 205–219.
52. Del Turco D, Woods AG, Gebhardt C, Phinney AL, Jucker M, et al. (2003) Comparison of commissural sprouting in the mouse and rat fascia dentata after entorhinal cortex lesion. Hippocampus 13: 685–699.
53. Stoppini L, Buchs PA, Muller D (1991) A simple method for organotypic cultures of nervous tissue. J Neurosci Methods 37: 173–182.
54. Keller S, Rupp C, Stoeck A, Runz S, Fogel M, et al. (2007) CD24 is a marker of exosomes secreted into urine and amniotic fluid. Kidney Int 72: 1095–1102.
